# Metabolic reprogramming of tumor-associated neutrophils in tumor treatment and therapeutic resistance

**DOI:** 10.3389/fcell.2025.1584987

**Published:** 2025-04-24

**Authors:** Jun Lin, Xian-Lu He, Wei-Wei Zhang, Chun-Fen Mo

**Affiliations:** ^1^ Department of General Surgery, Second Affiliated Hospital of Chengdu Medical College, China National Nuclear Corporation 416 Hospital, Chengdu, China; ^2^ Department of Immunology, School of Basic Medical Sciences, Chengdu Medical College, Chengdu, China; ^3^ School of Public Health, Chengdu Medical College, Chengdu, China

**Keywords:** tumor-associated neutrophils, tumor microenvironment, metabolic reprogramming, therapeutic resistance, immunotherapy

## Abstract

Tumor-associated neutrophils (TANs), pivotal immune cells within the tumor microenvironment (TME), exhibit dual potential in both pro- and anti-tumorigenic effects. These cells display remarkable heterogeneity and plasticity within the TME, adapting to hypoxic and nutrient-deprived conditions through metabolic reprogramming while critically influencing tumor progression, metastasis, and immune evasion. The metabolic reprogramming of TANs not only modulates their functional phenotypes but also reshapes tumor biological behaviors and therapeutic responses by regulating metabolic intermediates and cellular interactions within the TME. Therefore, elucidating the mechanisms underlying TANs metabolic reprogramming has significant implications for deciphering the molecular basis of tumorigenesis, identifying novel therapeutic targets, and optimizing immunotherapeutic strategies. This review systematically summarizes current knowledge regarding metabolic reprogramming mechanisms of TANs in the TME and their impact on tumor progression. We particularly focus on: 1) TAN-specific alterations in glucose, lipid, and amino acid metabolism within the TME; 2) Emerging immunotherapeutic strategies targeting TANs metabolic pathways; 3) Recent advances in understanding TAN-mediated immune evasion and therapy resistance. Furthermore, this review discusses potential challenges and corresponding solutions in targeting TANs metabolic reprogramming for therapeutic intervention, aiming to provide novel insights for advancing cancer immunotherapy.

## Graphical Abstract

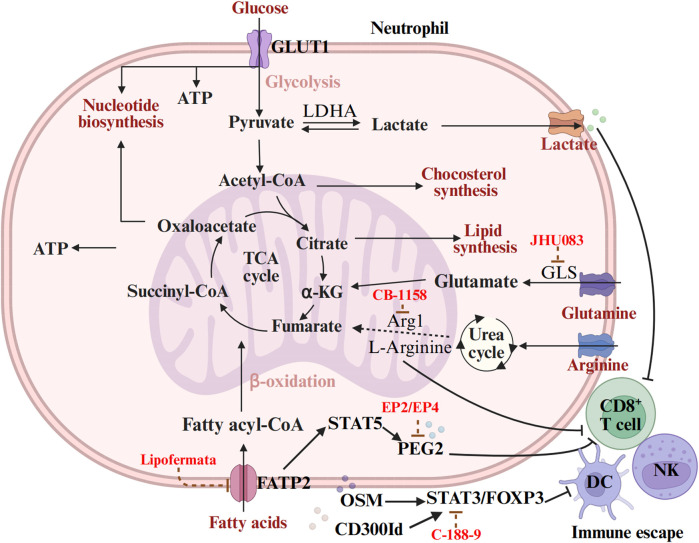

## 1 Introduction

Neutrophils are pivotal components of the innate immune system, playing critical roles in phagocytosis and bactericidal activity, antigen presentation, inflammatory responses, tissue remodeling, and homeostasis (Mihlan et al., 2024; Speaks et al., 2024; Wu et al., 2024). Neutrophils exhibit remarkable heterogeneity, with phenotypic diversity primarily determined by their developmental maturity, ranging from immature precursor neutrophils in the bone marrow to mature neutrophils in the peripheral blood circulation (Guo et al., 2024; Bai et al., 2025; He et al., 2025). Furthermore, neutrophils show high plasticity, differentiating into various phenotypes depending on the specific tissue microenvironments, which leads to distinct characteristics and regulatory functions in both physiological and pathological processes (Dong et al., 2024; Wang et al., 2024). Owing to their pronounced heterogeneity and plasticity, tumor-associated neutrophils (TANs) play a significant role in establishing cancer immunosuppression, and a high infiltration ratio of TANs is strongly correlated with poor prognosis in cancer patients (Lin et al., 2024; Schmidt et al., 2024; Tumbath et al., 2024; Teo et al., 2025). Consequently, TANs represent both a barrier to the development of novel anticancer immunotherapies and promising therapeutic targets.

During tumorigenesis and progression, malignant cells continuously adapt their metabolic patterns to secure sufficient nutrients for self-renewal and proliferation within the hypoxic and nutrient-deprived tumor microenvironment (TME), leading to alterations in metabolites (Ugolini et al., 2025). These metabolic abnormalities not only induce phenotypic and functional modifications in TANs but also drive their “metabolic reprogramming,” enabling them to exert immunosuppressive functions that facilitate tumor progression and metastasis (Pan et al., 2024). Therefore, an in-depth investigation of TANs metabolic reprogramming, coupled with targeted interventions to modulate TANs metabolic pathways and restore their antitumor functions, may emerge as a novel strategy for cancer immunotherapy.

In this review, we aim to discuss the principal metabolic pathways of TANs within the TME and explore immunotherapeutic strategies targeting these pathways, thereby providing new insights for advancing cancer immunotherapy.

## 2 TANs phenotype in TME

### 2.1 Origin and recruitment of TANs

**FIGURE 1:**
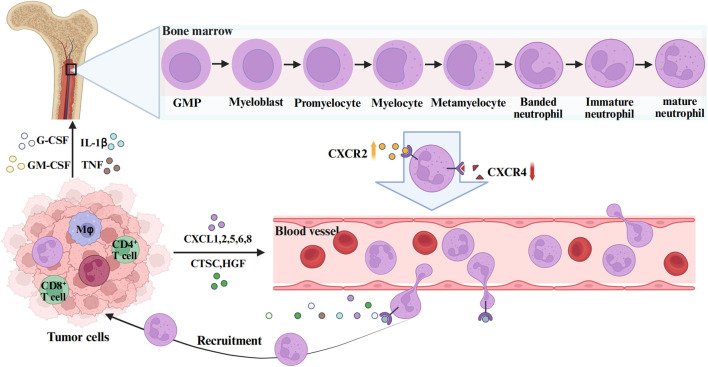
The formation and function of TANs. GMP is the upstream progenitor of all neutrophils during bone marrow hematopoiesis. Myeloblasts are adjacent downstream cells that subsequently differentiate successively into Promyelocytes myelocytes, metamyelocytes, and banded neutrophils, eventually becoming mature neutrophils. During this process, the expression of the neutrophil surface receptor CXCR4 decreases and CXCR2 increases, mediating the release of neutrophils into the peripheral blood. In the TME, tumor cells and stromal cells secrete CXC chemokine ligands (CXCL1, 2, 5, 6, 8), G-CSF, GM-CSF, IL-1β, TNF-α and other cytokines to mediate the recruitment and mobilization of neutrophils. The figure was created with BioRender.

TANs refer to neutrophils recruited to tumor tissues under the action of chemokines in the TME (Shaul and Fridlender, 2019). The formation of TANs mainly involves three stages: proliferation and maturation of neutrophils in the bone marrow, release into the peripheral blood circulation, and chemotactic migration to the TME (Figure 1).

All neutrophils originate from multipotent granulocyte-monocyte progenitors (GMPs) in the bone marrow, subsequently progressing through developmental stages including myeloblasts, promyelocytes, band cells, and segmented neutrophils to ultimately form mature neutrophils (Hidalgo et al., 2019). Mature neutrophils are released from the bone marrow into peripheral blood circulation in response to granulocyte colony-stimulating factor (G-CSF) (Furze and Rankin, 2008). Two critical chemokine receptors expressed on neutrophil surfaces are CXC chemokine receptor 4 (CXCR4) and CXC chemokine receptor 2 (CXCR2), where CXCR4 primarily governs neutrophil bone marrow homing, while CXCR2 and its ligands coordinate with G-CSF to mediate neutrophil release (Carnevale et al., 2023; Raftopoulou et al., 2022). During neutrophil maturation, upregulated CXCR2 expression facilitates their egress into the peripheral circulation. Following entry into the bloodstream, neutrophils exhibit widespread distribution and rapidly extravasate to inflammatory foci (Radtke and Voehringer, 2023). They subsequently execute immune effector functions through phagocytosis, degranulation, reactive oxygen species (ROS) generation, and neutrophil extracellular traps (NETs) formation (Shafqat et al., 2023). Post-inflammation resolution, residual neutrophils undergo two primary fates: a subset undergoes programmed cell death, while others demonstrate reverse transmigration back into the vasculature or re-entering peripheral circulation (Kraus and Gruber, 2021).

During tumorigenesis, cancer cells and associated stromal cells secrete substantial quantities of cytokines (e.g., IL-17A, TGF-β), chemokines (e.g., CXCL1, CXCL2), growth factors (e.g., G-CSF, GM-CSF), as well as lipids, which recruit a large population of neutrophils (SenGupta et al., 2021). These infiltrating neutrophils, exhibiting heterogeneous maturation states, migrate into the TME where a subset undergoes differentiation into TANs(Maiorino et al., 2022). Some studies propose that neutrophil-to-TAN conversion may initiate during the bone marrow phase (Engblom et al., 2017). Beyond bone marrow-derived TANs, the spleen, a reservoir of monocytes, also contributes to TAN populations within the TME (Cortez-Retamozo et al., 2012). However, whether phenotypic distinctions or differential recruitment mechanisms exist between bone marrow- and spleen-derived TANs remains uncertain, warranting further investigation.

### 2.2 Classification and polarization of TANs

TANs represent a heterogeneous subset of neutrophils characterized by remarkable plasticity, leading to continuous refinement of their classification system over time. Previous studies have demonstrated that neutrophils in cancer patients and tumor-bearing murine models can be stratified into two density-based subtypes: high-density neutrophils (HDNs) (Sagiv et al., 2015; Partouche et al., 2024), which possess anti-tumor properties, and low-density neutrophils (LDNs) (Hsu et al., 2019; Valadez-Cosmes et al., 2021), which exhibit immunosuppressive properties. During early tumorigenesis, HDNs predominate and demonstrate enhanced phagocytic capacity while mediating tumor cytotoxicity, thereby eliciting a systemic anti-tumor response. However, with tumor progression, the proportion of LDNs progressively increases. Notably, HDNs can undergo TGFβ-dependent differentiation into LDNs, resulting in diminished cytotoxic efficacy against neoplastic cells (Sagiv et al., 2015).

In 2007, researchers identified a population of myeloid cells with immunosuppressive properties in cancer patients, designated as myeloid-derived suppressor cells (MDSCs). These cells were further classified into two subtypes: polynuclear myeloid-derived suppressor cells (PMN-MDSCs) and monocytic myeloid-derived suppressor cells (M-MDSCs) (Zhou et al., 2018). Both subsets exhibit immature and immunosuppressive characteristics. Neutrophils demonstrate a close association with PMN-MDSCs, sharing common precursor cell origins, analogous differentiation pathways, and phenotypic similarities. In murine models, neutrophils display the phenotype CD11 b^+^Ly6G^+^Ly6Clow, while in humans, they are characterized as CD14^−^CD11 b^+^CD15^+^CD66 b^+^(Shaul and Fridlender, 2019).

Similar to tumor-associated macrophages (TAMs), TANs exhibit both pro- and anti-tumor potential within the TME and undergo functional polarization (Sica and Mantovani, 2012). In 2009, researchers inspired by TAM classification paradigms, categorized TANs into anti-tumor (N1) and pro-tumor (N2) subtypes (Fridlender et al., 2009). While this dichotomous framework historically facilitated TANs research, emerging evidence from multi-omics technologies increasingly demonstrate its insufficiency in capturing the phenotypic and functional diversity of TANs. Jaillon et al. subsequently proposed a refined classification system based on phenotypic heterogeneity, stratifying TANs into four subsets: immature neutrophils (NI), anti-tumor (N1), pro-tumor (N2), and interferon-stimulated gene signature-enriched neutrophils (NISG) (Health Commission Of The People’s Republic Of China, 2022). However, with the continuous discovery of novel TANs functional phenotypes, current classification criteria urgently require refinement to reflect their evolving biological complexity.

**FIGURE 2:**
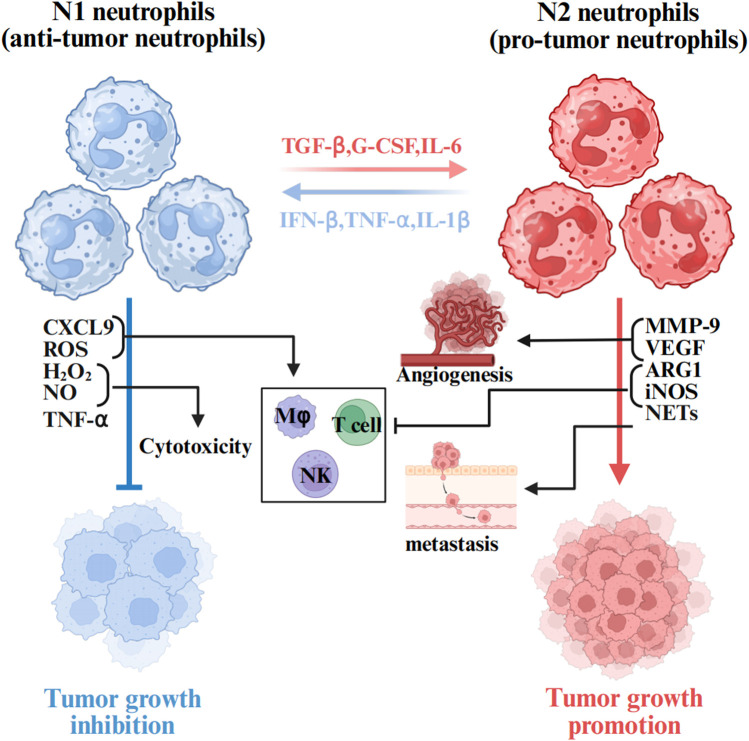
The role of TANs in the tumor microenvironment. Neutrophils are plastic cells that when exposed to cues within the TME are polarized toward a tumor inhibiting (N1) or tumor promoting (N2) type. Anti-tumor neutrophils mediate direct tumor cytotoxicity *via* mechanisms including ROS, NO, H_2_O_2_,TNF-α and CXCL9. Upon the activation of N2 by TGFβ, various effectors are secreted to induce pathological angiogenesis (MMPs and VEGF) and cell migration (NETs), contributing thus to the inhibition of the anti-tumor immune response (ARG1 and iNOS). The figure was created with BioRender.

The functional role of TANs within the TME is not definite. TANs undergo bidirectional phenotypic conversion between anti-tumor (N1) and pro-tumor (N2) phenotypes through polarization processes, a functional plasticity that is dynamically regulated by various cytokines and signaling pathways in the TME (Cortez-Retamozo et al., 2012). Within the TME, TANs polarization toward N1 or N2 phenotypes are governed by the balance between interferon-β (IFN-β) and transforming growth factor-β (TGF-β) signaling (Figure 2).

IFN-β induces N1 polarization of TANs. Early tumorigenesis, N1-polarized neutrophils capture and kill hepatocellular carcinoma (HCC) cells by releasing NETs and directly targeting tumor cells through mediators such as ROS, hydrogen peroxide (H_2_O_2_), nitric oxide (NO), and tumor necrosis factor-alpha (TNF-α), thereby exhibiting anti-tumor activity (Ohms et al., 2020; Siwicki and Pittet, 2021). Furthermore, the N1 phenotype enhances T-cell responses by integrating neutrophil and antigen-presenting cell properties, thus promoting anti-tumor immunity.

However, tumor progression may trigger phenotypic switching to N2 polarization driven by cytokines such as TGF-β and G-CSF. N2-polarized neutrophils are characterized by protumorigenic factor expression, including arginase 1 (Arg1), matrix metalloproteinase 9 (MMP-9), and vascular endothelial growth factor (VEGF). These mediators collectively promote angiogenesis, facilitate tumor cell migration/invasion, and establish an immunosuppressive niche *via* “immunosuppressive switch” mechanisms that support tumor progression (Quail et al., 2017; Carus et al., 2013). Notably, TGF-β serves as a master regulator of this phenotypic shift, and its pharmacological inhibitor SM16 effectively blocks N1-to-N2 polarization and modulates TAN functionality, thereby demonstrating promising antitumor therapeutic potential (Fridlender et al., 2009).

However, the TANs polarization is not confined within the TME. Researchers demonstrated in a murine breast cancer model that tumor-derived G-CSF induces myeloid differentiation reprogramming in the bone marrow, thereby increasing the number of TME-infiltrating N2-polarized TANs with T-cell suppressive functions (Giese et al., 2019). G-CSF also prolongs the half-life of TANs. This phenotypic transition associated with TANs polarization is accompanied by dynamic alterations in the secretome profile, including cytokines and chemokines (Antuamwine et al., 2023).

## 3 Metabolic reprogramming of neutrophils in the TME

The metabolic reprogramming of neutrophils involves multiple metabolic pathways, primarily including glycolysis, fatty acid metabolism, and amino acid metabolism. These metabolic pathways not only affects the functional phenotypes of neutrophils themselves but also exerts profound impacts on the biological behavior of tumors and therapeutic responses by modulating metabolites and cellular interactions in the TME. To elucidate these mechanisms, we have summarized the distinct regulators involved in these metabolic processes and their roles in regulating neutrophil functions (Table 1).

**TABLE 1:** Summary of distinct regulators (genes, proteins, etc.) involved in metabolisms and regulating neutrophil functions.

Metabolic pathways	Cancer types	Metabolites	Regulators	Function	References
Glucose metabolism	HCC	-	CXCL2, CXCL8	Facilitate the recruitment of neutrophils to the tumor site and promote the release of the OSM	Peng et al. (2020)
	Colorectal cancer	Lactate	LDHA, GPR37	Increase neutrophil recruitment within the microenvironment of CRLM	Zhou et al. (2023)
	Lung cancer	Lactate	GLUT1	enhance the efficacy of radiotherapy	Ancey et al. (2021)
	Breast cancer	-	PKM2, STAT5	Weaken T cell anti-tumor activity	Wang et al. (2023c)
	Gastric cancer	-	SLIT2	Promote tumor metastasis and induce neutrophils to polarize towards the tumor-promoting N2-type	Zhang et al. (2024)
	HCC	Lactate	LDHA, STAT3	Impair the function of cytotoxic T cells and promote neutrophils expressing PD-L1 and releasing the OSM	He et al. (2024)
	Colorectal cancer	Lactate	SPI1,SPIB	Activate the glycolytic and facilitate tumor development	Wang et al. (2021)
	Breast cancer	-	ENO1	Promote the proliferation and metastasis of cancer cells	Ou et al. (2021)
TCA	Breast cancer	Oxalate	HAO1	Facilitate the establishment of the pre-metastatic niche and induce the formation of NETs	Zeng et al. (2022)
	Ovarian cancer	α-KGDC	DLST	Reduction of oxidative phosphorylation	Udumula et al. (2021)
	Breast cancer	Aconitate	Acod1	Decrease TIN density, inhibit metastasis and enhance the anti-tumor T cell immune response	Zhao et al. (2023)
Lipid metabolism	Breast cancer	27-HC	LXR,CYP27A1	Promote angiogenesis and lung metastasis	Ma et al. (2020)
	Breast cancer	Fatty acid	ATGL	Enhance the lipid accumulation and promote metastasis	Li et al. (2020)
	Breast cancer	Cholesterol	ASPP2	Promote the formation of NETs	Tang et al. (2022)
	HCC	Acetyl-CoA	CXCL1, CXCR2	Inhibit NETs formation to reduce metastasis	Tang et al. (2022)
	Breast cancer	Oxygen sterols	LXR, CXCR2	Promote tumor growth through enhanced neovascularization and immunosuppression	Raccosta et al. (2013)
	Prostate cancer	Ether lipid	FAS	Maintain functionality of membranes	Lodhi et al. (2015)
Lipid metabolism	Colorectal cancer	Ether lipid	ECI2	Inhibits the production of ether lipids and diminish neutrophil recruitment and the formation of NETs	Chen et al. (2024)
	Lung cancers	FATP2/PGE2	STAT5	Diminish lipid accumulation, reduce ROS and block the immunosuppressive activity of MDSCs	Veglia et al. (2019)
Amino acid metabolism	melanoma	Glutamate	STAT3	Diminish the tumor-killing capacity and Shift from N1 to N2	Xiong et al. (2022a)
	Glioma	Arginine	ARG-1	Inhibit neutrophil activation and induce polarization of neutrophils to the N2 phenotype	Tsai et al. (2023)
	NSCLC	Arginine	ANXA2	Induce Arg1 expression and inhibit T cell viability	Zhang et al. (2022a)
	Breast cancer	Arginine	PAD4/PADI4	Decrease the tumor growth and reduce lung metastasis	Shi et al. (2020)
	Lung cancer	PGE2	IDO1	Inhibit CD8+ T cell function and promote tumor growth	Schafer et al. (2016)
	HCC	Serine	PHGDH	Promote neutrophil recruitment	Zhu et al. (2023)

### 3.1 Glucose metabolism

#### 3.1.1 Glycolysis

Glycolysis is intricately linked to the recruitment of TANs and the process of tumorigenesis (Wang et al., 2023b). Specifically, TANs infiltrate tumor sites in response to various chemical inducers, including cytokines, chemokines, and pro-inflammatory signals (Shaul and Fridlender, 2019; Que et al., 2022). Research indicates that the glycolytic switch in the tumor-infiltrating core solo cells mediate the production of CXCL2 and CXCL8 through the PFKFB3-NF-κB signaling pathway. This process facilitates the recruitment of neutrophils to the tumor site and promotes the release of the metastasis-promoting factor Oncostatin M (OSM) (Peng et al., 2020). In the context of colorectal cancer (CRC), the G Protein-Coupled Receptor 37 (GPR37) receptor enhances the expression of lactate dehydrogenase A (LDHA) and increases glycolytic activity by activating the Hippo signaling pathway. This activation results in significantly elevated levels of lactylation of H3K18la. This epigenetic modification leads to the upregulation of CXCL1 and CXC chemokine ligand 5 (CXCL5), ultimately increasing neutrophil recruitment within the microenvironment of CRC liver metastases (CRLM) (Zhou et al., 2023). These findings suggest that tumor cells regulate the expression of neutrophil chemokines through metabolic-epigenetic coupling, thereby establishing a vicious cycle that promotes metastasis.

Glycolytic metabolic reprogramming of TAN not only supports their survival and function but also directly contributes to key pro-tumor processes, such as angiogenesis and tumor metastasis. For instance, in human psoriasis, the glycolytic metabolism of CXCR4hi neutrophils undergo a significant shift, which enhances vascular permeability and exacerbates skin inflammatory responses (Rodriguez-Rosales et al., 2021). Concurrently, *in vitro* experiments demonstrated that CXCR4hi neutrophils exhibit increased capabilities in the formation of NETs, phagocytosis, degranulation, and the overexpression of pro-inflammatory cytokines and chemokines. This phenomenon is closely linked to their metabolic shift towards glycolysis and lactate release, which, in turn, facilitates disease progression by promoting vascular permeability and tissue remodeling (Chen et al., 2023). In HCC, tumor-infiltrating neutrophils also experience a tumor-induced metabolic shift towards glycolysis and the pentose phosphate pathway, which promotes the formation of NETs in a ROS-dependent manner. The formation of NETs not only enhances cancer cell migration but also downregulates tight junction proteins between endothelial cells, thereby disrupting vascular barrier function and promoting tumor infiltration and metastasis (Jiang et al., 2022). Overall, TANs form a metabolic synergy with cancer cells through glycolytic metabolic reprogramming, thereby constructing a pre-invasive TME.

Studies have revealed a close link between the hyperactivated glycolytic activity of neutrophils and their promotion of tumor function. In a mouse model of lung adenocarcinoma, TANs exhibited a significant increase in GLUT1 expression and glucose metabolism. Further investigations indicated that the deletion of GLUT1 accelerated the renewal of neutrophils within tumors and reduced the expression of SiglecF in TANs. More importantly, in the absence of GLUT1 expression, tumor growth was slowed, and the efficacy of radiotherapy was significantly enhanced (Ancey et al., 2021). Additionally, Wang and colleagues identified a heterogeneous protumor neutrophil subpopulation closely associated with poor prognosis in the pancreatic ductal adenocarcinoma (PDAC) TME through single-cell RNA sequencing technology (Wang et al., 2023b). This subpopulation is characterized by an abnormal enhancement of glycolytic activity, which further amplifies the tumor-promoting function of neutrophils. In breast cancer, studies have also revealed the immunosuppressive function of glycolytic neutrophils. Specifically, glycolytic neutrophils that accumulate in the spleen induce T cell non-responsiveness by disrupting pyruvate kinase M2 (PKM2) and its regulatory effect on signal transducer and activator of transcription 5(STAT5), thereby weakening their anti-tumor activity (Wang et al., 2023c). This suggests that glycolytic neutrophils not only directly promote tumor progression but also indirectly support tumor growth by inhibiting the anti-tumor immune response. In summary, the glycolytic activity of neutrophils plays a significant protumor role across various tumor types. However, further research into the heterogeneity of glycolytic neutrophils in different tumor microenvironments and their specific regulatory mechanisms are warranted.

The glycolytic process in tumor cells plays a crucial regulatory role in the phenotype and function of neutrophils. For instance, Zhang and colleagues demonstrated that gastric cancer cells induce neutrophils to polarize towards the tumor-promoting N2-type through the reprogramming of glucose metabolism (Zhang et al., 2024). These N2-type polarized neutrophils transport miR-4745-5p/3911 *via* exosomes, which inhibit the expression of Slit Guidance Ligand 2(SLIT2) in gastric cancer cells, thereby promoting tumor metastasis. Similarly, in HCC, serum amyloid A (SAA) activates the LDHA/STAT3 pathway to induce glycolysis, leading to neutrophils expressing PD-L1 and releasing the OSM(He et al., 2024). Consequently, the function of cytotoxic T cells is significantly impaired, further indicating that tumor cells not only regulate neutrophil function through metabolic reprogramming but also indirectly influence anti-tumor immune responses. TAN also impact mitochondrial function in hepatoma cells through various mechanisms. For example, during cancer progression, the speed and distance of mitochondrial movement in neutrophils are increased, alongside elevated rates of oxidative phosphorylation and glycolysis, resulting in increased adenosine production. Collectively, these changes regulate neutrophil migration behavior (Fu et al., 2023). In mouse models of early cancer, the rates of oxidative phosphorylation and glycolysis in bone marrow neutrophils are enhanced, leading to increased ATP production and improved spontaneous migration capacity. This enhanced migratory ability is regulated by autocrine ATP signaling mediated by purinergic receptors (Patel et al., 2018).

Neutrophil-derived exosomes play a crucial role in tumor progression by modulating the metabolic processes of cancer cells, particularly glycolysis. Recent studies have increasingly demonstrated specific mechanisms through which exosomes contribute to tumor development by delivering bioactive molecules, including mRNAs, miRNAs, and piRNAs. For instance, research has shown that exosomes produced by neutrophils can transfer *Salmonella* pathogenic island 1 (SPI1) mRNA to colorectal cancer cells, thereby activating the glycolytic pathway and facilitating tumor development (Wang et al., 2021). C5aR1^+^ neutrophils stimulate the glycolytic process in breast cancer cells through exosome-mediated regulation of Enolase 1 (ENO1) m6A methylation. Specifically, the ERK1/2-WTAP-dependent m6A methylation modification enhances the stability of ENO1 and its glycolytic activity, thereby providing metabolic support for the proliferation and metastasis of breast cancer cells (Ou et al., 2021). These studies underscore the pivotal role of neutrophil-derived exosomes in cancer glycolysis. By delivering molecules such as mRNA and miRNAs, exosomes not only directly regulate the metabolic state of cancer cells but also influence the tumor-promoting microenvironment by affecting stem cell characteristics and metastatic potential. However, despite these findings illuminate the significant role of exosomes, their specific regulatory mechanisms across different tumor types and microenvironments require further investigation.

#### 3.1.2 Tricarboxylic acid cycle

Circulating neutrophils primarily depend on glycolysis as their preferred metabolic strategy. However, the metabolic pathways of the neutrophil tricarboxylic acid (TCA) cycle can vary significantly under different nutritional, metabolic, and pathological conditions, particularly within the tumor microenvironment. These metabolic changes are crucial in tumorigenesis, progression, and the formation of the premetastatic niche (PMN). For instance, research indicates that hydroxyoxidase 1 (HAO1), the rate-limiting enzyme for oxalate synthesis, is significantly upregulated in mouse alveolar epithelial cells harboring metastatic breast cancer cells, resulting in abnormal accumulation of oxalate in lung tissue. This metabolic alteration facilitates the establishment of the pre-metastatic niche by activating NADPH oxidase, which induces the formation of NETs. Furthermore, the accumulation of oxalate in the lungs directly enhances the proliferation of metastatic cancer cells through the activation of the MAPK signaling pathway. Notably, pharmacological inhibition of HAO1 effectively blocked the lung oxalate accumulation induced by primary cancer, thereby significantly reducing lung metastasis of breast cancer. This finding not only elucidates the pivotal role of HAO1 in PMN formation but also suggests a potential strategy for anti-metastatic therapy that targets metabolic reprogramming (Zeng et al., 2022). Dihydrolipoamide succinyl transferase (DLST) is a crucial subunit of the α-ketoglutarate dehydrogenase complex (α-KGDC) within the TCA cycle. It has been identified as a tumor-induced gene whose expression is significantly elevated in myeloid cells. The inhibitory effect of DLST results in a reduction of oxidative phosphorylation in these myeloid cells, along with a decrease in the expression and function of immunosuppressive markers (Udumula et al., 2021).

Additional studies have demonstrated that the metabolic reprogramming of tumor-infiltrating neutrophils (TINs) also significantly contributes to the metastatic process. For example, Zhao et al. reported the induced expression of aconitate decarboxylase 1 (Acod1) in TINs and its critical role in inhibiting ferroptosis and maintaining TIN viability. Specifically, Acod1 regulates redox balance through its metabolite itaconate, thereby safeguarding TINs from ferroptosis. Experimental evidence showed that the ablation of Acod1 not only decreased TIN density but also significantly inhibited breast cancer metastasis while enhancing the anti-tumor T cell immune response. Importantly, mice lacking Acod1 exhibited more substantial anti-metastatic effects in response to immune checkpoint blockade therapy, indicating that Acod1 may serve as a potential target for combined immunotherapy (Zhao et al., 2023).

### 3.2 Lipid metabolism

#### 3.2.1 Fatty acid oxidation and lipid metabolism

Metabolic adaptation in neutrophils extends beyond glycolysis to encompass fatty acid oxidation and oxidative phosphorylation. Notably, in glucose-constrained environments, neutrophils can fulfill their metabolic requirements and sustain the production of ROS through fatty acid oxidation, which is essential for their immune functionality. For instance, in a 4T1 transplantable breast tumor model, a glucose-constrained environment induces immature c-Kit^+^neutrophil subpopulations to depend on mitochondrial fatty acid oxidation. This metabolic shift not only enhances ROS production but also contributes to T cell inhibition, NETosis, and the development of liver metastasis (Hsu et al., 2019; Rice et al., 2018). These findings suggest that the adaptive response of neutrophils under metabolic stress may significantly influence their immune function and their role within the tumor microenvironment by altering their metabolic pathways.

In recent years, research on neutrophil lipid metabolism and tumor progression has primarily concentrated on the role of neutrophils in cancer metastasis. As early as the late 1980s, it was discovered that the intravenous injection of cancer-laden rodents, along with neutrophils, significantly increased the incidence of lung metastasis (Pekarek et al., 1995). Subsequently, Ma and Baek et al. demonstrated that the activation of the IL-17/G-CSF signaling pathway or the accumulation of cholesterol metabolite 27-hydroxycholesterol (27-HC) can significantly elevate the number of circulating neutrophils, thus promoting angiogenesis and immunosuppression, which accelerates lung metastasis in breast cancer (Ma et al., 2020; Baek et al., 2017; Coffelt et al., 2015). Furthermore, Li et al. revealed that lung mesenchymal cells inhibit adipose triglyceride lipase (ATGL) activity in neutrophils through both prostaglandin E2-dependent and independent mechanisms, leading to lipid accumulation in neutrophils and ultimately promoting lung metastasis in breast cancer (Li et al., 2020). Another study indicated that leukotrienes produced by neutrophils selectively expand subpopulations of cancer cells with high tumor formation potential, facilitating their colonization of distant tissues. Notably, inhibiting the leukotriene-producing enzyme arachidonate 5-lipoxygenase (Alox5) through genetic or pharmacological interventions can abolish the pro-metastatic activity of neutrophils, thereby reducing the transfer of breast cancer cells to the lungs (Wculek and Malanchi, 2015). Similarly, in mouse breast cancer cell line 4T1 and human breast cancer cell line MDA-MB-231, depletion of ASPP2 triggers *de novo* cholesterol biosynthesis, which in turn promotes the formation of NETs *in vitro*, a process that regulates lung metastasis in breast cancer (Tang et al., 2022). Tyagi et al. indicate that long-term exposure to nicotine promotes the formation of premetastatic niches for breast cancer cells in the lungs. This process involves the release of lipid carrier-2 and the recruitment of tumor-promoting N2-neutrophils. Notably, low doses of rhodiola can effectively inhibit the polarization of nicotine-induced neutrophils to the N2 phenotype, while enhancing the N1 phenotype by reducing the expression of activated STAT3 (Tyagi et al., 2021). In summary, neutrophils play a crucial role in each step of the metastatic cascade, which includes the formation of premetastases, the escape of cancer cells from primary tumors, entry into blood and/or lymphovascular systems, survival in circulation, extravasation to distant organs, and the growth of metastases.

#### 3.2.2 Lipid metabolites

Metabolic abnormalities in tumor cells not only sustain their malignant phenotype but also significantly influence the tumor microenvironment by secreting metabolites or competing with immune cells for nutrients (Kaymak et al., 2021; Elia and Haigis, 2021). For instance, Pan et al. discovered that the accumulation of acetyl-CoA can induce the epigenetic activation of CXCL1 in hepatocellular carcinoma cells, thereby recruiting TANs and forming NETs that promote cancer cell migration and metastasis. This finding suggests that inhibiting CXCL1-CXCR2 signaling and NET formation may serve as an effective strategy to reduce liver cancer metastasis, particularly in patients with elevated acetyl-CoA levels, which opens new avenues for potential therapeutic targets (Pan et al., 2024). Additionally, Baek et al. reported that the cholesterol metabolite 27-hydroxycholesterol can modulate breast cancer metastasis by reshaping the tumor microenvironment and facilitating the recruitment of immune cells, such as neutrophils and γδT cells, to metastatic niches (Baek et al., 2017). Further investigations have revealed that cholesterol-derived metabolite oxygen sterol plays a crucial role in various malignant tumors, including breast cancer, prostate cancer, colon cancer, and cholangiocarcinoma (Kloudova et al., 2017). Raccosta et al. demonstrated that tumor-derived oxygen sterols recruit pre-tumor neutrophils in a liver X receptor (LXR)-independent and CXCR2-dependent manner, thereby promoting tumor growth through enhanced neovascularization and immunosuppression (Raccosta et al., 2013). In summary, these studies elucidate how tumor metabolic abnormalities regulate the function of immune cells, particularly neutrophils, by altering metabolites in the microenvironment, thereby facilitating tumor progression and metastasis. Consequently, interventions targeting these metabolic pathways and their associated signaling cascades may offer innovative strategies for tumor treatment.

#### 3.2.3 Ether lipid metabolism

Disorders in ether lipid metabolism and the formation of NETs have recently been identified as potential risk factors for tumorigenesis and progression. Research indicates that abnormal ether lipid metabolism may be closely linked to tumor evolution and metastasis (Dahabieh et al., 2018). For instance, Lodhi et al. demonstrated that the complete loss of fatty acid synthase (FAS) in adult mice results in the disruption of intestinal barrier function and a reduction in neutrophil count, ultimately leading to mortality. Notably, the decrease in neutrophil numbers appears to be associated with the effects of peroxisome-derived ether lipid membrane content, contributing to increased endoplasmic reticulum stress and apoptosis. Additionally, the loss of FAS is correlated with a significant and specific impairment in granulocyte production within the bone marrow, indicating that FAS is crucial for the survival of mature neutrophils. These findings suggest that the FAS/PexRAP axis may play a pivotal role in maintaining the lipid composition and functionality of neutrophil membranes. Importantly, the targeted inhibition of lipidogenic enzymes may influence the disease trajectory associated with neutrophils, thereby highlighting a previously underappreciated connection between endogenous lipid metabolism and inflammation (Lodhi et al., 2015). Another study demonstrated that lipid metabolism-related gene enoyl Coenzyme A δ-isomerase 2 (ECI2) can inhibit the production of ether lipids by restricting the peroxisomal localization of the ether lipid metabolism rate-limiting enzyme alkyl-glycerone phosphate synthase (AGPS). This inhibition subsequently reduces the expression of interleukin 8 (IL-8), leading to diminished neutrophil recruitment and the formation of extracellular neutrophil traps, thereby inhibiting colorectal cancer (Chen et al., 2024). In summary, these studies not only elucidate the close relationship between disorders of ether lipid metabolism and neutrophil function but also provide a novel perspective on their roles in tumorigenesis, progression, and metastasis. Consequently, interventions targeting ether lipid metabolism may represent a significant strategy for future cancer treatment.

#### 3.2.4 Lipoprotein metabolism

PMN-MDSCs are activated neutrophils that emerge under pathological conditions and play a critical role in modulating the immune response to cancer. These cells are implicated in the poor efficacy of cancer treatments and are closely associated with unfavorable clinical outcomes. Numerous studies have demonstrated that lipid accumulation in cancer is observed across the entire population of macrophages, dendritic cells (DCs), and MDSCs, correlating with their immunosuppressive activity (Al-Khami et al., 2017; O'Neill and Pearce, 2016; Cubillos-Ruiz et al., 2015). In various mouse tumor models, increased fatty acid uptake and fatty acid oxidation (FAO) in tumor-infiltrating MDSCs (T-MDSCs) have been linked to enhanced mitochondrial mass, upregulation of essential FAO enzymes, and elevated oxygen consumption. These findings indicate that inhibiting fatty acid oxidation may modulate the immunosuppressive functions of myelogenic inhibitory cells and improve the efficacy of cancer therapies (Hossain et al., 2015). Further research has revealed that tumor cell-derived GM-CSF regulates the overexpression of fatty acid transporter 2 (FATP2) in PMN-MDSCs through the activation of the STAT5 transcription factor. Notably, FATP2 facilitates the immunosuppressive activity of PMN-MDSCs in tumors by mediating the uptake of arachidonic acid and the synthesis of prostaglandin E2 (PGE2) (Veglia et al., 2019). Additionally, the inhibition of FATP2 expression in MDSCs using adipocetoma drugs can diminish lipid accumulation, reduce ROS, and block the immunosuppressive activity of MDSCs, thereby inhibiting tumor growth (Adeshakin et al., 2021).

PMN-MDSCs contribute to tumor immune tolerance and can lead to the failure of tumor immunotherapy. For instance, Shi et al. demonstrated that the feedback loop between the FATP2 and receptor-interacting protein kinase 3 (RIPK3) pathways in PMN-MDSCs significantly enhances the synthesis of PGE2, which in turn promotes the inhibitory activity of PMN-MDSCs on CD8^+^ T cell function and facilitates bladder cancer tumor growth (Shi et al., 2022). Additionally, Shi et al. discovered that bladder cancer (BCa)-derived exosome circRNA_0013936 upregulates FATP2 *via* the circRNA_0013936/miR-320a/JAK2 pathway and downregulates RIPK3 through the circRNA_0013936/miR-301b-3p/CREB1 pathway in PMN-MDSCs, resulting in a significant inhibition of CD8^+^ T cell function and, consequently, tumor immunity (Shi et al., 2024).

These studies elucidate the mechanisms by which PMN-MDSCs regulate their immunosuppressive functions through lipid metabolism, specifically fatty acid oxidation and FATP2-mediated PGE2 synthesis, as well as through signaling pathways involving RIPK3 and circRNA-related pathways. Consequently, targeting these metabolic and signaling pathways may offer novel strategies to enhance the efficacy of cancer immunotherapy.

### 3.3 Amino acid metabolism

In addition to glycolysis, tricarboxylic acid cycle, and fatty acid metabolism, the amino acid metabolism of TANs is also reprogrammed during tumor development. Research indicates that tumor cells systematically alter intracellular amino acid metabolism and extracellular amino acid distribution to meet their proliferation requirements, leading to metabolic reprogramming and remodeling of the TME.

#### 3.3.1 Glutamine and glutamate metabolism

In the absence of glucose supply, newly generated neutrophils can derive energy through the hydrolysis of glutamine. Specifically, glutamine is first converted to glutamic acid, which is subsequently metabolized into α-ketoglutaric acid within the mitochondria; this compound is utilized for oxidative phosphorylation in the TCA cycle. Oxidative phosphorylation (OXPHOS) facilitates the generation of key intermediate metabolites necessary for energy production (Sadiku et al., 2021). Further investigations in a mouse model of hypoxic acute lung injury revealed that neutrophils could eliminate extracellular proteins associated with lung injury, thereby promoting central carbon metabolism and utilizing the derived glutamine to meet their energy requirements (Watts et al., 2021). Additionally, in the ovarian cancer cell line ID8, TANs rely on glutamine as their primary fuel for OXPHOS, which helps sustain their immunosuppressive effects (Udumula et al., 2021).

Glutamate released by tumor cells can promote the phosphorylation of STAT3 in neutrophils, transitioning them from a tumor-killing phenotype to an immunosuppressive phenotype, a process believed to represent a shift from N1 to N2 (Xiong et al., 2022a). Further research has demonstrated that substantial release of glutamate by tumor cells diminishes the tumor-killing capacity of neutrophils both *in vitro* and *in vivo*. Specifically, elevated levels of pSTAT3, RAB10, and ARF4 mediated by glutamate is associated with the immunosuppressive phenotypes of neutrophils within the TME. Notably, riluzole, a glutamate release inhibitor, significantly inhibits tumor growth by restoring the cytotoxic capabilities of neutrophils and reducing glutamate secretion from tumor cells (Xiong et al., 2022b). Additionally, neutrophils exhibit phenotypic heterogeneity and can perform either anti-metastatic or pro-metastatic functions. For instance, Hsu et al.'s study indicates that cancer-derived G-CSF is essential for mobilizing immature low-density neutrophils (iLDNs), which promote liver metastasis. Further investigations have revealed that iLDNs depend on the catabolism of glutamate and proline to sustain mitochondria-dependent metabolism in the absence of glucose, thereby facilitating sustained NETosis, which enhances the liver transfer of breast cancer (Hsu et al., 2019).

#### 3.3.2 Arginine metabolism

The tumor microenvironment is typically characterized by numerous immunosuppressive mechanisms (Grobben, 2024). Among these, myeloid cells play a significant role in inhibiting anti-tumor immunity through various metabolic pathways, such as the degradation of L-arginine. The concentration of L-arginine is primarily regulated by ARG1 and inducible nitric oxide synthase (NOS2) (Canè et al., 2023) Research has demonstrated that high expression and activity of ARG1 are present in circulating and tumor-infiltrating myeloid cells across different cancer types, with granulocyte-like myelogenic inhibitory cells (G-MDSCs) identified as their main source (Steggerda et al., 2017; Zhang et al., 2017). Under hypoxic conditions, acrolein produced by glioma cells inhibits neutrophil activation *in vitro*, induces polarization of neutrophils to the N2 phenotype, and promotes the production of ARG-1. Furthermore, studies indicate that ARG-1 inhibits AKT activity by directly interacting with the Cys310 residue of AKT, thereby facilitating glioma progression (Tsai et al., 2023). Additionally, arginase-1 is upregulated in N2 neutrophils and has been shown to impair T-cell function. The degradation of arginine can also lead to cell cycle arrest in T cells, preventing their replication (Oberg et al., 2019). For instance, in human non-small cell lung cancer (NSCLC), Annexin A2 (ANXA2) signals through the TLR2/MYD88 axis in neutrophils, inducing ARG1 mRNA expression through amino acid depletion, which in turn inhibits T-cell viability (Zhang et al., 2022b).

Peptidyl arginine deiminase 4 (PAD4/PADI4) is an enzyme involved in post-translational modification that converts protein arginine or monomethyl arginine to citrulline. Research indicates that the PAD4-mediated hypercitrullination reaction in neutrophils results in the release of nuclear chromatin, which forms a chromatin network known as NETs. For instance, mouse breast cancer 4T1 cells that express elevated levels of PADI4 can release cancer extracellular chromatin networks (CECN) both *in vitro* and *in vivo*. Notably, the deletion of Padi4 completely abolished CECN formation in 4T1 cells, decreased the tumor growth rate in allograft models, and reduced lung metastasis associated with 4T1 breast cancer (Shi et al., 2020). Further investigations have demonstrated that chloroquine (CQ) and hydroxychloroquine (HCQ) can inhibit the formation of NETs by blocking PAD4 enzyme activity, consequently impeding tumor growth and metastasis (Ivey et al., 2023). Additionally, in the 4T1 orthotopic mouse model, the PAD4 inhibitor compound 28 disrupted the PAD4-H3cit-NET signaling pathway, thereby inhibiting the growth of solid tumors and lung metastatic nodules. Importantly, compound 28 also enhances the tumor immune microenvironment by modifying the neutrophil phenotype, increasing the proportion of dendritic and M1 macrophages, and decreasing the number of myeloid-derived suppressor cells (Zhu et al., 2024). Similarly, Huangqin Decoction (HQD) has been shown to inhibit the onset of colitis-related carcinoma by regulating PAD4-dependent extracellular traps (Pan et al., 2022). Concurrently, glycyrrhizic acid mitigates the formation of extracellular traps in neutrophils by inhibiting PAD4, thus improving outcomes in colitis-related colorectal cancer (Chen et al., 2025).

TANs upregulate the expression of indoleamine 2,3-dioxygenase 1 (IDO1) in cancer cells by secreting PGE2 in patients with urothelial bladder cancer (UBC), thereby inhibiting CD8^+^ T cell function (Ouyang et al., 2024b). Similarly, host IDO1 has been shown to play a significant role in promoting tumor growth, MDSC accumulation, and the expression of PD-1 on CD8^+^ T cells in the Lewis lung cancer model (Schafer et al., 2016).

#### 3.3.3 Serine metabolism

Serine metabolism plays a crucial role in regulating neutrophil function. Specifically, phosphoglycerate dehydrogenase (PHGDH) is a key enzyme in the serine biosynthesis pathway. Studies have demonstrated that in liver cancer cells, the aspartate kinase-chorismate mutase-tyrosine aminotransferase (ACT) domain of PHGDH binds to nuclear cMyc, forming a transactivation axis involving PHGDH, p300, cMyc, and AF9, which drives the gene expression of chemokines CXCL1 and IL8. Subsequently, CXCL1 and IL8 promote neutrophil recruitment and enhance the filtration of TAM in the liver, thereby facilitating liver cancer progression (Zhu et al., 2023). Furthermore, cathepsin G, a neutrophil-derived serine protease, plays various roles in the tumor microenvironment. For instance, cathepsin G induces cell migration, activates insulin-like growth factor 1 (IGF-1), increases E-cadherin-mediated intercellular adhesion and cancer cell aggregation, and promotes the entry of cancer cells into blood vessels (Morimoto-Kamata and Yui, 2017). In summary, serine metabolism and its associated enzymes significantly influence tumor progression by regulating neutrophil function and the immune response within the tumor microenvironment.

## 4 Potential therapeutic strategies targeting TANs’ metabolic reprogramming

Cancer cell-derived stromal cells, which may reside within the TME, contribute to carcinogenic processes, including cancer cell proliferation, invasion, and metastasis. As key regulatory components of the TME, TANs exert pleiotropic functions that support cancer cell survival, enhance angiogenesis, and facilitate tumor dissemination and metastasis. Notably, TANs have emerged as potential therapeutic targets due to their capacity to either pro-or anti-tumor progression through context-dependent immunomodulatory effects. To this end, we summarize potential therapeutic strategies targeting the metabolic reprogramming of TANs (Table 2).

**TABLE 2:** Neutrophil targeting therapeutic strategies.

Pathways	Targets	Drugs	Cancer types	Beneficial Anti-cancer effects	Reference
Glucose metabolism	MIF	-	Breast cancer	Inhibit glycolysis, decrease Treg and increase CD8+ T cells and macrophages	Yan et al. (2024)
	HIF-1α	-	Colorectal cancer	Inhibit the immunosuppressive function of MDSCs	Zhang et al. (2022c)
	ACOD1	-	Breast cancer	Alleviate T cell immunosuppression and enhance the ICB by promoting ferroptosis	Kim et al. (2022)
Lipid metabolism	FATP2	Lipofermata		Inhibit the uptake of triglycerides and the synthesis of PGE2	Wellenstein and de Visser (2019)
	PGE2	Celecoxib	Breast cancer	Improve the efficacy of adoptive T cell immunotherapy	Ouyang et al. (2024a)
Amino acid metabolism	Glutaminase	JHU083	Breast cancer	Reduce the recruitment of G-CSF and MDSCs and inhibit glycolysis	Oh et al. (2020)
	ARG1	CB-1158	Glioma	Mitigate T cell inhibition	Steggerda et al. (2017)

Reprogramming of energy metabolism plays a pivotal role in cancer progression and immune surveillance. Studies have demonstrated that macrophage migration inhibitory factor (MIF), secreted by breast cancer stem cells (BCSCs), activates the WNT/β-catenin signaling pathway to upregulate c-MYC-mediated expression of aldolase C, thereby promoting glycolytic metabolism in tumor cells. Furthermore, targeted inhibition of MIF not only suppresses glycolysis but also significantly reduces tumor growth and metastasis. Importantly, MIF depletion alters the immunological composition of the TME, leading to increased intratumoral lytic CD8^+^T cells and proinflammatory macrophages, while concomitantly decreasing regulatory T cells and TANs. These findings collectively suggest that MIF targeting could enhance the therapeutic efficacy of immune checkpoint blockade (ICB) therapy in triple-negative breast cancer (TNBC). Therefore, MIF emerges as a critical regulator of metabolic reprogramming and immunosuppression in breast cancer, representing a promising therapeutic strategy to improve ICB-based immunotherapy outcomes (Yan et al., 2024). Another study revealed that inhibiting lactic acid production or disrupting the function of HIF-1α, a critical regulator of neutrophils, can impede tumor progression (Yang et al., 2020). For example, *C. tropicalis* enhances the immunosuppressive function of MDSCs through the Syk-PKM2-HIF-1α-glycolysis signaling axis, thereby promoting the development of colorectal cancer (CRC). Thus, these findings establish the Syk-PKM2-HIF-1α-glycolytic signaling axis as a potential therapeutic target for CRC (Zhang et al., 2022c). Further, ferroptosis in TANs contributes to immunosuppressive TME remodeling (Kim et al., 2022). Recent studies have demonstrated that the expression of metabolic enzyme aconitate decarboxylase 1 (ACOD1) is significantly upregulated in both murine and human TANs, which is governed by the GM-CSF-JAK/STAT5-C/EBPβ signaling pathway. Notably, loss of ACOD1 in TANs has been shown to enhance the efficacy of ICB therapy through the induction of ferroptosis. For example, in ICB-treated tumor-bearing ACOD1−/− mice, the proportion of CD8^+^ and CD4^+^ T cells was markedly increased. Consequently, ACOD1 deficiency in TANs promotes their survival within the TME, contributes to tumor-associated immunosuppression, and ultimately diminishes the therapeutic efficacy of ICB (Zhao et al., 2023).

Modulation of lipid metabolism in TANs can profoundly affect their metabolic and functional profiles. Specifically, selective inhibition of fatty acid oxidation abolishes TANs’ immunosuppressive capacity, leading to marked attenuation of tumor growth (Bodac and Meylan, 2021). Further investigations revealed that fatty acid transport protein 2 (FATP2) is specifically upregulated in human and murine PMN-MDSCs. Mechanistically, GM-CSF in TME activates STAT5, which binds to the slc27a2 promoter region to upregulate FATP2 expression. Upregulated FATP2 enhances PMN-MDSC-mediated CD8^+^ T cell suppression through increased glycerol trioleate uptake and prostaglandin E2 (PGE2) synthesis, thereby promoting tumor progression (Veglia et al., 2019). Additionally, lipofermata (a selective FATP2-mediated fatty acid transport inhibitor) combined with anti-PD-1 therapy augments ICB efficacy, improving patient survival and prognosis (Wellenstein and de Visser, 2019). Recent studies have demonstrated that a leucine-rich diet can significantly enhance the effectiveness of immunotherapy (Wu et al., 2024). Simultaneously, blockade of EP2/EP4 receptors suppresses the Ptgs2/PGE2 axis, reducing neutrophil immunosuppression, alleviating pulmonary metastasis in breast cancer, and enhancing adoptive T cell therapy outcomes (Gong et al., 2023). Notably, celecoxib-mediated COX-2/PGE2 inhibition similarly strengthens ICB therapeutic responses (Ouyang et al., 2024a). In liver cancer, Yang et al. identified that NPC1 (niemann-pick C1 protein, a cholesterol intracellular transporter) exhibits elevated mRNA and protein expression in hepatocellular carcinoma (HCC) tissues, which correlates with poor clinical prognosis. Mechanistic analysis revealed that NPC1 overexpression promotes HCC progression by enhancing neutrophil recruitment, positioning NPC1 as a promising biomarker and therapeutic target for HCC (Yang et al., 2024).

Given the metabolic dependency of both tumor cells and TANs on glutamine, therapeutic strategies targeting glutamine metabolic pathways have emerged as particularly critical. Systemic administration of the glutaminase inhibitor JHU083 in 4T1 breast tumor-bearing mice resulted in reduced recruitment of G-CSF and MDSCs, along with induction of apoptosis in both intratumoral and circulating MDSC populations (Oh et al., 2020). Furthermore, this agent activated T cell activation and survival while inhibiting glycolysis and oxidative metabolism in several syngeneic tumor models (Leone et al., 2019). Notably, TANs promote the transcriptional expression of ARG1, leading to arginine depletion and subsequent suppression of T cell function, a critical mechanism underlying tumor immune evasion (Zhang et al., 2022a). Clinically, inhibitors targeting immunosuppressive neutrophil factors such as ARG1, nitric oxide (NO), and reactive oxygen species (ROS) have been investigated (Faget et al., 2021). The ARG1 inhibitor CB-1158 has demonstrated the potential to convert immunosuppressive tumor microenvironments into proinflammatory states, thereby alleviating T cell suppression and reducing tumor growth. Combination of CB-1158 with other immunotherapies may therefore enhance patient outcomes through synergistic modulation of the TME (Steggerda et al., 2017).

Modulating metabolic products generated from tumor cell or neutrophil metabolism can enhance neutrophil anti-tumor activity. Research has demonstrated that extracellular acidic pH (pH 6.8) may upregulate spermidine/spermine N1-acetyltransferase 1 (SAT1) expression, thereby promoting the accumulation of the tumor growth metabolite N1-acetylspermidine. Immunological analysis revealed that SAT1 inhibition reduces neutrophil recruitment to tumors, consequently impairing angiogenesis and tumor growth, thereby identifying a novel metabolic target for cancer immunotherapy (Kato et al., 2023). Additional studies identified that tumor-secreted nicotinamide phosphoribosyltransferase (NAMPT) reprograms CD10+ALPL + neutrophils in the TME through the NTRK1 pathway, maintaining their immature state and suppressing maturation/activation. This process induces “irreversible” CD8^+^ T cell exhaustion, enabling tumor evasion of anti-PD-1 therapy’s anticipated efficacy (Meng et al., 2023).

## 5 TANs’ metabolic reprogramming in tumor immune evasion and therapeutic resistance

**FIGURE 3:**
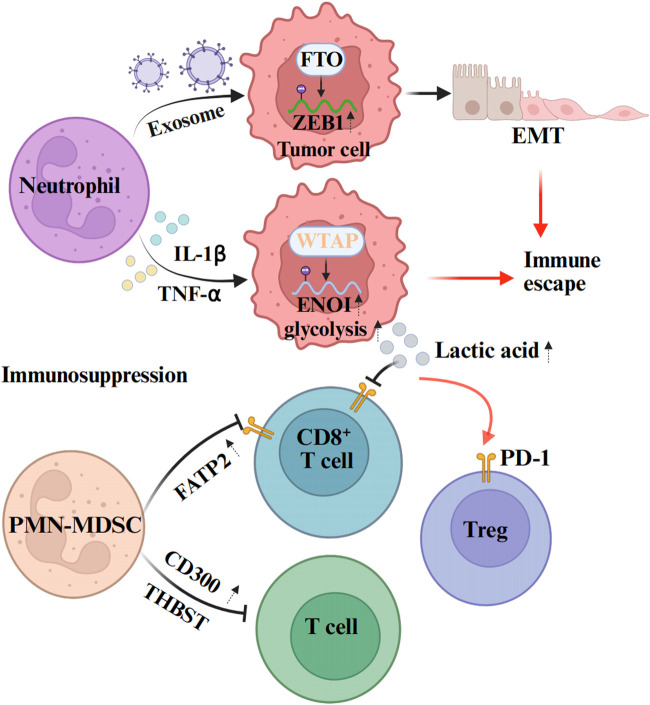
Mechanisms of neutrophil-mediated tumor immunotherapy resistance. Neutrophils can affect the expression of epigenetic molecules in tumor cells through exosomes, and promote the immune escape of tumors. From the aspect of promoting the formation of immunosuppressive microenvironment, neutrophils mainly inhibit the activation and proliferation of immune cells through some cytokines and signaling pathways, and promote their injury, apoptosis or transformation to immunosuppressive phenotype. The figure was created with BioRender.

The tolerance of tumors to immunotherapy is closely related to the formation of an immunosuppressive TME. Neutrophils play essential roles (mediator release and surface immune checkpoint molecules) through interactions with immune cells. Consequently, we have summarized the mechanisms by which neutrophils mediate tumor immunotherapy resistance to provide a clearer understanding of this complex process (Figure 3).

Tumor resistance is evolutionarily driven by genetic and/or epigenetic changes within cancer cells (Goenka et al., 2023). For example, investigations of neutrophils isolated from tumor tissue and peripheral blood of patients have demonstrated that neutrophil-derived exosome piRNA-17560 can enhance the expression of fat mass and obesity-associated proteins (FTO) in breast cancer cells. Further research has revealed that the upregulation of FTO expression increases the stability of zinc finger E-box-binding homeobox protein 1 (ZEB1) and elevates ZEB1 transcript levels by reducing m6A RNA methylation. This cascade of changes ultimately leads to the epithelial-mesenchymal transformation (EMT) of tumor cells, thereby promoting chemotherapy resistance in breast cancer cells (Ou et al., 2022). Moreover, emerging evidence indicates a close functional interplay between glucose metabolic reprogramming in the TME and immune evasion. Notably, Ou et al. indicate that c5ar1-positive neutrophils modulate tumor glucose metabolism through WTAP-dependent m6A methylation of α-enolase (ENOI) (Ou et al., 2021). Collectively, these findings suggest that targeted interventions against neutrophils, as well as the regulation of epigenetic molecule-mediated tumor immune resistance, may represent a potential therapeutic strategy. These findings collectively suggest that targeted interventions against neutrophil functions, combined with epigenetic modulation of immune-resistant pathways, may represent promising therapeutic strategy for overcoming tumor drug resistance.

TANs metabolic reprogramming plays a crucial role in tumor immune evasion. It can establish an immunosuppressive microenvironment through metabolic alterations, interfere with the recruitment and functions of immune cells, and also affect the metabolic states of other immune cells within the TME. Studies have demonstrated that TANs preferentially activate glycolytic pathways in the TME, leading to massive lactate production. Lactate promotes the nuclear translocation of nuclear factor of activated T-cells 1 (NFAT1), a master regulator of immune cell proliferation, thereby enhancing PD-1 expression in regulatory T cells (Tregs) while suppressing PD-1 expression in effector T cells. This mechanism results in the activation of PD-1-expressing Tregs during PD-1 blockade therapy, ultimately causing immunotherapy failure (Kumagai et al., 2022). Furthermore, lactic acid within the TME serves as a potent functional inhibitor of T cell and NK cell. Accumulation of this metabolic byproduct disrupts immune surveillance capabilities mediated by these lymphocyte subsets, ultimately promoting tumor immune evasion (Brand et al., 2016). In addition to glucose metabolic derivatives, lipid metabolic mediators in neutrophils also play a regulatory role in tumor immunity. For example, neutrophils can upregulate the expression of fatty acid transporter protein 2 (FATP2), thereby enhancing immunosuppression and promoting tumor progression. Experimental evidence indicates that pharmacological inhibitior of FATP2 abolishes neutrophil-mediated immunosuppressive activity, and its combination with immune checkpoint inhibitors (ICIs) effectively blocks tumor progression (Veglia et al., 2019). These findings elucidate the critical roles of neutrophil-derived metabolic mediators (e.g., lactic acid and FATP2) in tumor immune escape and therapeutic resistance. Consequently, targeting these neutrophil-associated metabolic pathways represent a promising therapeutic strategy to overcome immune resistance in cancer.

Furthermore, recent investigations have identified that, despite playing a crucial role in tumor progression and therapeutic resistance, polymorphonuclear myeloid-derived suppressor cells (PMN-MDSCs) present significant challenges for targeted interventions due to their myeloid origin and shared surface markers with classical neutrophils (Li et al., 2023). However, recent studies have revealed that CD300 expression is upregulated in PMN-MDSCs, where it functions through the STAT3-S100A8/A9 axis to suppress T cell activation and tumor immune resistance. Thus, antibody-mediated CD300 inhibition was shown to attenuate tumor progression and exhibit synergistic efficacy when combined with anti-PD-1 treatment (Wang et al., 2023a). Furthermore, PMN-MDSCs can induce cytotoxic T-cell exhaustion and colorectal cancer resistance to immune checkpoint inhibitors (ICIs) through THBS1 (Omatsu et al., 2023). These findings suggest that neutrophils (including PMN-MDSCs) may exploit surface immune checkpoint molecules to suppress T-cell immunity, thereby promoting tumor resistance to immunotherapy. Consequently, targeting immune checkpoint ligands on PMN-MDSCs, such as CD300 and THBS1, could represent a novel therapeutic strategy to overcome immune resistance in cancer. In summary, the TANs metabolic reprogramming remodels the TME *via* modulating extracellular acidification, immune cell functionalities, and tumor metabolic plasticity, thereby constructing a multidimensional immunosuppressive network that serves as a critical mechanism for tumor immune evasion.

## 6 Conclusion

The metabolic reprogramming of TANs, primarily including glycolysis, fatty acid metabolism, and amino acid metabolism, plays a pivotal role in tumor therapy. TANs not only modulate their own functional phenotypes but also affect tumor biological behavior and therapeutic responses *via* regulating metabolites and cellular interactions in the TME. Specifically, glycolytic reprogramming in TANs supports their survival and functional execution while directly participating in key pro-tumorigenic processes, including angiogenesis and metastasis. Fatty acid metabolic reprogramming enables TANs to sustain ROS production under glucose-deprived conditions, thereby impacting their immune functions. Amino acid metabolic reprogramming induces immune suppression through the depletion of essential amino acids while promoting cancer progression. Furthermore, TANs’ metabolic reprogramming is closely associated with tumor therapeutic resistance, such as facilitating immune evasion and chemo-resistance through epigenetic regulation and metabolite modulation.

Although existing studies have revealed the critical importance of TANs’ metabolic reprogramming in tumor progression, several limitations and challenges remain: First, the heterogeneity and plasticity of TANs creates complex regulatory mechanisms for their metabolic reprogramming, and current research fail to comprehensively elucidate their specific mechanisms across different tumor types and microenvironments. Second, most investigations rely on animal models or *in vitro* experiments, lacking clinical validation that restricts translational potential. Additionally, therapeutic strategies targeting TANs’ metabolic reprogramming remain in their infancy, requiring further exploration into effective interventions to restore their anti-tumor functions.

Future research should deepen the investigation into molecular mechanisms underlying TANs’ metabolic reprogramming, particularly their tumor-type and microenvironment-specific roles. More clinical studies are warranted to validate the relationship between TANs’ metabolic reprogramming and therapeutic outcomes/drug resistance, thereby informing novel treatment strategies. Concurrently, therapeutic approaches targeting TANs’ metabolic pathways, such as the inhibition of specific metabolic enzymes or signaling pathways, require optimization and clinical potential evaluation. Exploration of synergistic combinations with existing immunotherapies to enhance TANs’ anti-tumor efficacy and overcome immune resistance is also crucial. Finally, investigating the interplay between TANs’ metabolic reprogramming and other immune cells within TME’s regulatory network will provide comprehensive insights into TANs’ roles in tumor progression, potentially yielding innovative strategies for more effective anticancer therapies.

## References

[B1] Adeshakin A. O. Liu W. Adeshakin F. O. Afolabi L. O. Zhang M. Zhang G. (2021). Regulation of ROS in myeloid-derived suppressor cells through targeting fatty acid transport protein 2 enhanced anti-PD-L1 tumor immunotherapy. Cell Immunol. 362, 104286. 10.1016/j.cellimm.2021.104286 33524739

[B2] AL-Khami A. A. Zheng L. Del Valle L. Hossain F. Wyczechowska D. Zabaleta J. (2017). Exogenous lipid uptake induces metabolic and functional reprogramming of tumor-associated myeloid-derived suppressor cells. Oncoimmunology 6, e1344804. 10.1080/2162402X.2017.1344804 29123954 PMC5665069

[B3] Ancey P.-B. Contat C. Boivin G. Sabatino S. Pascual J. Zangger N. (2021). GLUT1 expression in tumor-associated neutrophils promotes lung cancer growth and resistance to radiotherapy. Cancer Res. 81, 2345–2357. 10.1158/0008-5472.CAN-20-2870 33753374 PMC8137580

[B4] Antuamwine B. B. Bosnjakovic R. Hofmann-Vega F. Wang X. Theodosiou T. Iliopoulos I. (2023). N1 versus N2 and PMN-MDSC: a critical appraisal of current concepts on tumor-associated neutrophils and new directions for human oncology. Immunol. Rev. 314, 250–279. 10.1111/imr.13176 36504274

[B5] Baek A. E. Yu Y. A. He S. Wardell S. E. Chang C. Y. Kwon S. (2017). The cholesterol metabolite 27 hydroxycholesterol facilitates breast cancer metastasis through its actions on immune cells. Nat. Commun. 8, 864. 10.1038/s41467-017-00910-z 29021522 PMC5636879

[B6] Bai M. Jin Y. Jin Z. Xie Y. Chen J. Zhong Q. (2025). Distinct immunophenotypic profiles and neutrophil heterogeneity in colorectal cancer. Cancer Lett. 616, 217570. 10.1016/j.canlet.2025.217570 39993650

[B7] Bodac A. Meylan E. (2021). Neutrophil metabolism in the cancer context. Semin. Immunol. 57, 101583. 10.1016/j.smim.2021.101583 34963565

[B8] Brand A. Singer K. Koehl G. E. Kolitzus M. Schoenhammer G. Thiel A. (2016). LDHA-associated lactic acid production blunts tumor immunosurveillance by T and NK cells. Cell Metab. 24, 657–671. 10.1016/j.cmet.2016.08.011 27641098

[B9] Canè S. Barouni R. M. Fabbi M. Cuozzo J. Fracasso G. Adamo A. (2023). Neutralization of NET-associated human ARG1 enhances cancer immunotherapy. Sci. Transl. Med. 15, eabq6221. 10.1126/scitranslmed.abq6221 36921034

[B10] Carnevale S. DI Ceglie I. Grieco G. Rigatelli A. Bonavita E. Jaillon S. (2023). Neutrophil diversity in inflammation and cancer. Front. Immunol. 14, 1180810. 10.3389/fimmu.2023.1180810 37180120 PMC10169606

[B11] Carus A. Ladekarl M. Hager H. Nedergaard B. S. Donskov F. (2013). Tumour-associated CD66b+ neutrophil count is an independent prognostic factor for recurrence in localised cervical cancer. Br. J. Cancer 108, 2116–2122. 10.1038/bjc.2013.167 23591202 PMC3670536

[B12] Chen Y. L. Xu B. Pan Z. F. Cai Y. P. Yang C. Y. Cao S. L. (2025). Glycyrrhizic acid reduces neutrophil extracellular trap formation to ameliorate colitis-associated colorectal cancer by inhibiting peptidylarginine deiminase 4. J. Ethnopharmacol. 341, 119337. 10.1016/j.jep.2025.119337 39788166

[B13] Chen J. Bai Y. Xue K. Li Z. Zhu Z. Li Q. (2023). CREB1-driven CXCR4hi neutrophils promote skin inflammation in mouse models and human patients. Nat. Commun. 14, 5894. 10.1038/s41467-023-41484-3 37736772 PMC10516899

[B14] Chen L. Dai P. Liu L. Chen Y. Lu Y. Zheng L. (2024). The lipid-metabolism enzyme ECI2 reduces neutrophil extracellular traps formation for colorectal cancer suppression. Nat. Commun. 15, 7184. 10.1038/s41467-024-51489-1 39169021 PMC11339366

[B15] Coffelt S. B. Kersten K. Doornebal C. W. Weiden J. Vrijland K. Hau C. S. (2015). IL-17-producing γδ T cells and neutrophils conspire to promote breast cancer metastasis. Nature 522, 345–348. 10.1038/nature14282 25822788 PMC4475637

[B16] Cortez-Retamozo V. Etzrodt M. Newton A. Rauch P. J. Chudnovskiy A. Berger C. (2012). Origins of tumor-associated macrophages and neutrophils. Proc. Natl. Acad. Sci. U. S. A. 109, 2491–2496. 10.1073/pnas.1113744109 22308361 PMC3289379

[B17] Cubillos-Ruiz J. R. Silberman P. C. Rutkowski M. R. Chopra S. Perales-Puchalt A. Song M. (2015). ER stress sensor XBP1 controls anti-tumor immunity by disrupting dendritic cell homeostasis. Cell 161, 1527–1538. 10.1016/j.cell.2015.05.025 26073941 PMC4580135

[B18] Dahabieh M. S. DI Pietro E. Jangal M. Goncalves C. Witcher M. Braverman N. E. (2018). Peroxisomes and cancer: the role of a metabolic specialist in a disease of aberrant metabolism. Biochim. Biophys. Acta Rev. Cancer 1870, 103–121. 10.1016/j.bbcan.2018.07.004 30012421

[B19] Dong Y. Kang Z. Zhang Z. Zhang Y. Zhou H. Liu Y. (2024). Single-cell profile reveals the landscape of cardiac immunity and identifies a cardio-protective Ym-1(hi) neutrophil in myocardial ischemia-reperfusion injury. Sci. Bull. (Beijing) 69, 949–967. 10.1016/j.scib.2024.02.003 38395651

[B20] Elia I. Haigis M. C. (2021). Metabolites and the tumour microenvironment: from cellular mechanisms to systemic metabolism. Nat. Metab. 3, 21–32. 10.1038/s42255-020-00317-z 33398194 PMC8097259

[B21] Engblom C. Pfirschke C. Zilionis R. Da Silva Martins J. Bos S. A. Courties G. (2017). Osteoblasts remotely supply lung tumors with cancer-promoting SiglecF(high) neutrophils. Science 358, eaal5081. 10.1126/science.aal5081 29191879 PMC6343476

[B22] Faget J. Peters S. Quantin X. Meylan E. Bonnefoy N. (2021). Neutrophils in the era of immune checkpoint blockade. J. Immunother. Cancer 9, e002242. 10.1136/jitc-2020-002242 34301813 PMC8728357

[B23] Fridlender Z. G. Sun J. Kim S. Kapoor V. Cheng G. Ling L. (2009). Polarization of tumor-associated neutrophil phenotype by TGF-beta: “N1” versus “N2” TAN. Cancer Cell 16, 183–194. 10.1016/j.ccr.2009.06.017 19732719 PMC2754404

[B24] Furze R. C. Rankin S. M. (2008). Neutrophil mobilization and clearance in the bone marrow. Immunology 125, 281–288. 10.1111/j.1365-2567.2008.02950.x 19128361 PMC2669132

[B25] Fu S. Deng H. Bertolini I. Perego M. Chen E. S. Sanseviero E. (2023). Syntaphilin regulates neutrophil migration in cancer. Cancer Immunol. Res. 11, 278–289. 10.1158/2326-6066.CIR-22-0035 36548516 PMC9991994

[B26] Giese M. A. Hind L. E. Huttenlocher A. (2019). Neutrophil plasticity in the tumor microenvironment. Blood 133, 2159–2167. 10.1182/blood-2018-11-844548 30898857 PMC6524564

[B27] Goenka A. Khan F. Verma B. Sinha P. Dmello C. C. Jogalekar M. P. (2023). Tumor microenvironment signaling and therapeutics in cancer progression. Cancer Commun. (Lond) 43, 525–561. 10.1002/cac2.12416 37005490 PMC10174093

[B28] Gong Z. Li Q. Shi J. Li P. Hua L. Shultz L. D. (2023). Immunosuppressive reprogramming of neutrophils by lung mesenchymal cells promotes breast cancer metastasis. Sci. Immunol. 8, eadd5204. 10.1126/sciimmunol.add5204 36800412 PMC10067025

[B29] Grobben Y. (2024). Targeting amino acid-metabolizing enzymes for cancer immunotherapy. Front. Immunol. 15, 1440269. 10.3389/fimmu.2024.1440269 39211039 PMC11359565

[B30] Guo R. Xie X. Ren Q. Liew P. X. (2024). New insights on extramedullary granulopoiesis and neutrophil heterogeneity in the spleen and its importance in disease. J. Leukoc. Biol. 117, qiae220. 10.1093/jleuko/qiae220 39514106

[B31] Health commission of the people's republic of china (2022). National guidelines for diagnosis and treatment of gastric cancer 2022 in China (English version). Chin. J. Cancer Res. 34, 207–237. 10.21147/j.issn.1000-9604.2022.03.04 35873885 PMC9273576

[B32] He M. Liu Y. Chen S. Deng H. Feng C. Qiao S. (2024). Serum amyloid A promotes glycolysis of neutrophils during PD-1 blockade resistance in hepatocellular carcinoma. Nat. Commun. 15, 1754. 10.1038/s41467-024-46118-w 38409200 PMC10897330

[B33] He W. Yan L. Hu D. Hao J. Liou Y. C. Luo G. (2025). Neutrophil heterogeneity and plasticity: unveiling the multifaceted roles in health and disease. MedComm 6, e70063, 10.1002/mco2.70063 39845896 PMC11751288

[B34] Hidalgo A. Chilvers E. R. Summers C. Koenderman L. (2019). The neutrophil life cycle. Trends Immunol. 40, 584–597. 10.1016/j.it.2019.04.013 31153737

[B35] Hossain F. AL-Khami A. A. Wyczechowska D. Hernandez C. Zheng L. Reiss K. (2015). Inhibition of fatty acid oxidation modulates immunosuppressive functions of myeloid-derived suppressor cells and enhances cancer therapies. Cancer Immunol. Res. 3, 1236–1247. 10.1158/2326-6066.CIR-15-0036 26025381 PMC4636942

[B36] Hsu B. E. TabarièS S. Johnson R. M. Andrzejewski S. Senecal J. LehuéDé C. (2019). Immature low-density neutrophils exhibit metabolic flexibility that facilitates breast cancer liver metastasis. Cell Rep. 27, 3902–3915.e6. 10.1016/j.celrep.2019.05.091 31242422

[B37] Ivey A. D. Matthew Fagan B. Murthy P. Lotze M. T. Zeh H. J. Hazlehurst L. A. (2023). Chloroquine reduces neutrophil extracellular trap (NET) formation through inhibition of peptidyl arginine deiminase 4 (PAD4). Clin. Exp. Immunol. 211, 239–247. 10.1093/cei/uxad005 36655514 PMC10038322

[B38] Jiang Z.-Z. Peng Z.-P. Liu X.-C. Guo H.-F. Zhou M.-M. Jiang D. (2022). Neutrophil extracellular traps induce tumor metastasis through dual effects on cancer and endothelial cells. OncoImmunology 11, 2052418. 10.1080/2162402X.2022.2052418 35309732 PMC8928819

[B39] Kato M. Maeda K. Nakahara R. Hirose H. Kondo A. Aki S. (2023). Acidic extracellular pH drives accumulation of N1-acetylspermidine and recruitment of protumor neutrophils. PNAS Nexus 2, pgad306. 10.1093/pnasnexus/pgad306 37822765 PMC10563787

[B40] Kaymak I. Williams K. S. Cantor J. R. Jones R. G. (2021). Immunometabolic interplay in the tumor microenvironment. Cancer Cell 39, 28–37. 10.1016/j.ccell.2020.09.004 33125860 PMC7837268

[B41] Kim R. Hashimoto A. Markosyan N. Tyurin V. A. Tyurina Y. Y. Kar G. (2022). Ferroptosis of tumour neutrophils causes immune suppression in cancer. Nature 612, 338–346. 10.1038/s41586-022-05443-0 36385526 PMC9875862

[B42] Kloudova A. Guengerich F. P. Soucek P. (2017). The role of oxysterols in human cancer. Trends Endocrinol. Metab. 28, 485–496. 10.1016/j.tem.2017.03.002 28410994 PMC5474130

[B43] Kraus R. F. Gruber M. A. (2021). Neutrophils-from bone marrow to first-line defense of the innate immune system. Front. Immunol. 12, 767175. 10.3389/fimmu.2021.767175 35003081 PMC8732951

[B44] Kumagai S. Koyama S. Itahashi K. Tanegashima T. Lin Y. T. Togashi Y. (2022). Lactic acid promotes PD-1 expression in regulatory T cells in highly glycolytic tumor microenvironments. Cancer Cell 40, 201–218.e9. 10.1016/j.ccell.2022.01.001 35090594

[B45] Leone R. D. Zhao L. Englert J. M. Sun I. M. Oh M. H. Sun I. H. (2019). Glutamine blockade induces divergent metabolic programs to overcome tumor immune evasion. Science 366, 1013–1021. 10.1126/science.aav2588 31699883 PMC7023461

[B46] Lin Q. Zong S. Wang Y. Zhou Y. Wang K. Shi F. (2024). Breast cancer-derived CAV1 promotes lung metastasis by regulating integrin α6β4 and the recruitment and polarization of tumor-associated neutrophils. Int. J. Biol. Sci. 20, 5695–5714. 10.7150/ijbs.94153 39494337 PMC11528463

[B47] Li P. Lu M. Shi J. Gong Z. Hua L. Li Q. (2020). Lung mesenchymal cells elicit lipid storage in neutrophils that fuel breast cancer lung metastasis. Nat. Immunol. 21, 1444–1455. 10.1038/s41590-020-0783-5 32958928 PMC7584447

[B48] Li X. Ke Y. Hernandez A. L. Yu J. Bian L. Hall S. C. (2023). Inducible nitric oxide synthase (iNOS)-activated Cxcr2 signaling in myeloid cells promotes TGFβ-dependent squamous cell carcinoma lung metastasis. Cancer Lett. 570, 216330. 10.1016/j.canlet.2023.216330 37524225 PMC10530117

[B49] Lodhi I. Wei X. Yin L. Feng C. Adak S. Abou-Ezzi G. (2015). Peroxisomal lipid synthesis regulates inflammation by sustaining neutrophil membrane phospholipid composition and viability. Cell Metab. 21, 51–64. 10.1016/j.cmet.2014.12.002 25565205 PMC4287274

[B50] Maiorino L. DaßLER-Plenker J. Sun L. Egeblad M. (2022). Innate immunity and cancer pathophysiology. Annu. Rev. Pathol. 17, 425–457. 10.1146/annurev-pathmechdis-032221-115501 34788549 PMC9012188

[B51] Ma L. Wang L. Nelson A. T. Han C. He S. Henn M. A. (2020). 27-Hydroxycholesterol acts on myeloid immune cells to induce T cell dysfunction, promoting breast cancer progression. Cancer Lett. 493, 266–283. 10.1016/j.canlet.2020.08.020 32861706 PMC7572761

[B52] Meng Y. Ye F. Nie P. Zhao Q. An L. Wang W. (2023). Immunosuppressive CD10(+)ALPL(+) neutrophils promote resistance to anti-PD-1 therapy in HCC by mediating irreversible exhaustion of T cells. J. Hepatol. 79, 1435–1449. 10.1016/j.jhep.2023.08.024 37689322

[B53] Mihlan M. Wissmann S. Gavrilov A. Kaltenbach L. Britz M. Franke K. (2024). Neutrophil trapping and nexocytosis, mast cell-mediated processes for inflammatory signal relay. Cell 187, 5316–5335.e28. 10.1016/j.cell.2024.07.014 39096902

[B54] Morimoto-Kamata R. Yui S. (2017). Insulin-like growth factor-1 signaling is responsible for cathepsin G-induced aggregation of breast cancer MCF-7 cells. Cancer Sci. 108, 1574–1583. 10.1111/cas.13286 28544544 PMC5543509

[B55] Oberg H. H. Wesch D. Kalyan S. Kabelitz D. (2019). Regulatory interactions between neutrophils, tumor cells and T cells. Front. Immunol. 10, 1690. 10.3389/fimmu.2019.01690 31379875 PMC6657370

[B56] Oh M. H. Sun I. H. Zhao L. Leone R. D. Sun I. M. Xu W. (2020). Targeting glutamine metabolism enhances tumor-specific immunity by modulating suppressive myeloid cells. J. Clin. Invest 130, 3865–3884. 10.1172/JCI131859 32324593 PMC7324212

[B57] Ohms M. MöLLER S. Laskay T. (2020). An attempt to polarize human neutrophils toward N1 and N2 phenotypes *in vitro* . Front. Immunol. 11, 532. 10.3389/fimmu.2020.00532 32411122 PMC7198726

[B58] Omatsu M. Nakanishi Y. Iwane K. Aoyama N. Duran A. Muta Y. (2023). THBS1-producing tumor-infiltrating monocyte-like cells contribute to immunosuppression and metastasis in colorectal cancer. Nat. Commun. 14, 5534. 10.1038/s41467-023-41095-y 37749092 PMC10520015

[B59] O'Neill L. A. Pearce E. J. (2016). Immunometabolism governs dendritic cell and macrophage function. J. Exp. Med. 213, 15–23. 10.1084/jem.20151570 26694970 PMC4710204

[B60] Ou B. Liu Y. Gao Z. Xu J. Yan Y. Li Y. (2022). Senescent neutrophils-derived exosomal piRNA-17560 promotes chemoresistance and EMT of breast cancer via FTO-mediated m6A demethylation. Cell Death Dis. 13, 905. 10.1038/s41419-022-05317-3 36302751 PMC9613690

[B61] Ou B. Liu Y. Yang X. Xu X. Yan Y. Zhang J. (2021). C5aR1-positive neutrophils promote breast cancer glycolysis through WTAP-dependent m6A methylation of ENO1. Cell Death and Dis. 12, 737. 10.1038/s41419-021-04028-5 PMC8313695 34312368

[B62] Ouyang Y. Zhong W. Xu P. Wang B. Zhang L. Yang M. (2024a). Tumor-associated neutrophils suppress CD8(+) T cell immunity in urothelial bladder carcinoma through the COX-2/PGE2/Ido1 Axis. Br. J. Cancer 130, 880–891. 10.1038/s41416-023-02552-z 38233491 PMC10912642

[B63] Ouyang Y. Zhong W. Xu P. Wang B. Zhang L. Yang M. (2024b). Tumor-associated neutrophils suppress CD8+ T cell immunity in urothelial bladder carcinoma through the COX-2/PGE2/Ido1 Axis. Br. J. Cancer 130, 880–891. 10.1038/s41416-023-02552-z 38233491 PMC10912642

[B64] Pan J. J. Xie S. Z. Zheng X. Xu J. F. Xu H. Yin R. Q. (2024). Acetyl-CoA metabolic accumulation promotes hepatocellular carcinoma metastasis via enhancing CXCL1-dependent infiltration of tumor-associated neutrophils. Cancer Lett. 592, 216903. 10.1016/j.canlet.2024.216903 38670307

[B65] Pan Z. Xie X. Chen Y. Pan S. Wu Z. Yang C. (2022). Huang Qin Decoction inhibits the initiation of experimental colitis associated carcinogenesis by controlling the PAD4 dependent NETs. Phytomedicine 107, 154454. 10.1016/j.phymed.2022.154454 36155218

[B66] Partouche S. Goldberg I. Halperin E. Atamna B. Shacham-Abulafia A. Shapira S. (2024). Interferon-α inhibits NET formation in neutrophils derived from patients with myeloproliferative neoplasms in a neutrophil sub-population-specific manner. Int. J. Mol. Sci. 25, 13473. 10.3390/ijms252413473 39769242 PMC11677445

[B67] Patel S. Fu S. Mastio J. Dominguez G. A. Purohit A. Kossenkov A. (2018). Unique pattern of neutrophil migration and function during tumor progression. Nat. Immunol. 19, 1236–1247. 10.1038/s41590-018-0229-5 30323345 PMC6195445

[B68] Pekarek L. A. Starr B. A. Toledano A. Y. Schreiber H. (1995). Inhibition of tumor growth by elimination of granulocytes. J. Exp. Med. 181, 435–440. 10.1084/jem.181.1.435 7807024 PMC2191807

[B69] Peng Z. P. Jiang Z. Z. Guo H. F. Zhou M. M. Huang Y. F. Ning W. R. (2020). Glycolytic activation of monocytes regulates the accumulation and function of neutrophils in human hepatocellular carcinoma. J. Hepatol. 73, 906–917. 10.1016/j.jhep.2020.05.004 32407813

[B70] Quail D. F. Olson O. C. Bhardwaj P. Walsh L. A. Akkari L. Quick M. L. (2017). Obesity alters the lung myeloid cell landscape to enhance breast cancer metastasis through IL5 and GM-CSF. Nat. Cell Biol. 19, 974–987. 10.1038/ncb3578 28737771 PMC6759922

[B71] Que H. Fu Q. Lan T. Tian X. Wei X. (2022). Tumor-associated neutrophils and neutrophil-targeted cancer therapies. Biochim. Biophys. Acta Rev. Cancer 1877, 188762. 10.1016/j.bbcan.2022.188762 35853517

[B72] Raccosta L. Fontana R. Maggioni D. Lanterna C. Villablanca E. J. Paniccia A. (2013). The oxysterol-CXCR2 axis plays a key role in the recruitment of tumor-promoting neutrophils. J. Exp. Med. 210, 1711–1728. 10.1084/jem.20130440 23897983 PMC3754872

[B73] Radtke D. Voehringer D. (2023). Granulocyte development, tissue recruitment, and function during allergic inflammation. Eur. J. Immunol. 53, e2249977. 10.1002/eji.202249977 36929502

[B74] Raftopoulou S. Valadez-Cosmes P. Mihalic Z. N. Schicho R. Kargl J. (2022). Tumor-mediated neutrophil polarization and therapeutic implications. Int. J. Mol. Sci. 23, 3218. 10.3390/ijms23063218 35328639 PMC8951452

[B75] Rice C. M. Davies L. C. Subleski J. J. Maio N. Gonzalez-Cotto M. Andrews C. (2018). Tumour-elicited neutrophils engage mitochondrial metabolism to circumvent nutrient limitations and maintain immune suppression. Nat. Commun. 9, 5099. 10.1038/s41467-018-07505-2 30504842 PMC6269473

[B76] Rodriguez-Rosales Y. A. Langereis J. D. Gorris M. A. J. VAN Den Reek J. Fasse E. Netea M. G. (2021). Immunomodulatory aged neutrophils are augmented in blood and skin of psoriasis patients. J. Allergy Clin. Immunol. 148, 1030–1040. 10.1016/j.jaci.2021.02.041 33745888

[B77] Sadiku P. Willson J. A. Ryan E. M. Sammut D. Coelho P. Watts E. R. (2021). Neutrophils fuel effective immune responses through gluconeogenesis and glycogenesis. Cell Metab. 33, 1062–1064. 10.1016/j.cmet.2021.03.018 33951466 PMC8102058

[B78] Sagiv J. Y. Michaeli J. Assi S. Mishalian I. Kisos H. Levy L. (2015). Phenotypic diversity and plasticity in circulating neutrophil subpopulations in cancer. Cell Rep. 10, 562–573. 10.1016/j.celrep.2014.12.039 25620698

[B79] Schafer C. C. Wang Y. Hough K. P. Sawant A. Grant S. C. Thannickal V. J. (2016). Indoleamine 2,3-dioxygenase regulates anti-tumor immunity in lung cancer by metabolic reprogramming of immune cells in the tumor microenvironment. Oncotarget 7, 75407–75424. 10.18632/oncotarget.12249 27705910 PMC5340181

[B80] Schmidt E. Distel L. Erber R. BüTTNER-Herold M. Rosahl M. C. Ott O. J. (2024). Tumor-associated neutrophils are a negative prognostic factor in early luminal breast cancers lacking immunosuppressive macrophage recruitment. Cancers (Basel) 16, 3160. 10.3390/cancers16183160 39335132 PMC11430230

[B81] Sengupta S. Hein L. E. Parent C. A. (2021). The recruitment of neutrophils to the tumor microenvironment is regulated by multiple mediators. Front. Immunol. 12, 734188. 10.3389/fimmu.2021.734188 34567000 PMC8461236

[B82] Shafqat A. Khan J. A. Alkachem A. Y. Sabur H. Alkattan K. Yaqinuddin A. (2023). How neutrophils shape the immune response: reassessing their multifaceted role in health and disease. Int. J. Mol. Sci. 24, 17583. 10.3390/ijms242417583 38139412 PMC10744338

[B83] Shaul M. E. Fridlender Z. G. (2019). Tumour-associated neutrophils in patients with cancer. Nat. Rev. Clin. Oncol. 16, 601–620. 10.1038/s41571-019-0222-4 31160735

[B84] Shi L. Yao H. Liu Z. Xu M. Tsung A. Wang Y. (2020). Endogenous PAD4 in breast cancer cells mediates cancer extracellular chromatin network formation and promotes lung metastasis. Mol. Cancer Res. 18, 735–747. 10.1158/1541-7786.MCR-19-0018 32193354 PMC7668292

[B85] Shi X. Pang S. Zhou J. Yan G. Gao R. Wu H. (2024). Bladder-cancer-derived exosomal circRNA_0013936 promotes suppressive immunity by up-regulating fatty acid transporter protein 2 and down-regulating receptor-interacting protein kinase 3 in PMN-MDSCs. Mol. Cancer., 23, 52. 10.1186/s12943-024-01968-2 38461272 PMC10924381

[B86] Shi X. Pang S. Zhou J. Yan G. Sun J. Tan W. (2022). Feedback loop between fatty acid transport protein 2 and receptor interacting protein 3 pathways promotes polymorphonuclear neutrophil myeloid-derived suppressor cells-potentiated suppressive immunity in bladder cancer. Mol. Biol. Rep. 49, 11643–11652. 10.1007/s11033-022-07924-x 36169895

[B87] Sica A. Mantovani A. (2012). Macrophage plasticity and polarization: *in vivo* veritas. J. Clin. Invest 122, 787–795. 10.1172/JCI59643 22378047 PMC3287223

[B88] Siwicki M. Pittet M. J. (2021). Versatile neutrophil functions in cancer. Semin. Immunol. 57, 101538. 10.1016/j.smim.2021.101538 34876331

[B89] Speaks S. Mcfadden M. I. Zani A. Solstad A. Leumi S. Roettger J. E. (2024). Gasdermin D promotes influenza virus-induced mortality through neutrophil amplification of inflammation. Nat. Commun. 15, 2751. 10.1038/s41467-024-47067-0 38553499 PMC10980740

[B90] Steggerda S. M. Bennett M. K. Chen J. Emberley E. Huang T. Janes J. R. (2017). Inhibition of arginase by CB-1158 blocks myeloid cell-mediated immune suppression in the tumor microenvironment. J. Immunother. Cancer 5, 101. 10.1186/s40425-017-0308-4 29254508 PMC5735564

[B91] Tang Q. Liang B. Zhang L. Li X. Li H. Jing W. (2022). Enhanced CHOLESTEROL biosynthesis promotes breast cancer metastasis via modulating CCDC25 expression and neutrophil extracellular traps formation. Sci. Rep. 12, 17350. 10.1038/s41598-022-22410-x 36253427 PMC9576744

[B92] Teo J. M. N. Chen Z. Chen W. Tan R. J. Y. Cao Q. Chu Y. (2025). Tumor-associated neutrophils attenuate the immunosensitivity of hepatocellular carcinoma. J. Exp. Med. 222, e20241442. 10.1084/jem.20241442 39636298 PMC11619716

[B93] Tsai H. C. Tong Z. J. Hwang T. L. Wei K. C. Chen P. Y. Huang C. Y. (2023). Acrolein produced by glioma cells under hypoxia inhibits neutrophil AKT activity and suppresses anti-tumoral activities. Free Radic. Biol. Med. 207, 17–28. 10.1016/j.freeradbiomed.2023.06.027 37414347

[B94] Tumbath S. Jiang L. Li X. Zhang T. Zahid K. R. Zhao Y. (2024). β-Lapachone promotes the recruitment and polarization of tumor-associated neutrophils (TANs) toward an antitumor (N1) phenotype in NQO1-positive cancers. Oncoimmunology 13, 2363000. 10.1080/2162402X.2024.2363000 38846085 PMC11155710

[B95] Tyagi A. Sharma S. Wu K. Wu S.-Y. Xing F. Liu Y. (2021). Nicotine promotes breast cancer metastasis by stimulating N2 neutrophils and generating pre-metastatic niche in lung. Nat. Commun. 12, 474. 10.1038/s41467-020-20733-9 33473115 PMC7817836

[B96] Udumula M. P. Sakr S. Dar S. Alvero A. B. Ali-Fehmi R. Abdulfatah E. (2021). Ovarian cancer modulates the immunosuppressive function of CD11b(+)Gr1(+) myeloid cells via glutamine metabolism. Mol. Metab. 53, 101272. 10.1016/j.molmet.2021.101272 34144215 PMC8267600

[B97] Ugolini A. De Leo A. Yu X. Scirocchi F. Liu X. Peixoto B. (2025). Functional reprogramming of neutrophils within the brain tumor microenvironment by hypoxia-driven histone lactylation. Cancer Discov. 10.1158/2159-8290.CD-24-1056 PMC12133432 40014923

[B98] Valadez-Cosmes P. Maitz K. Kindler O. Raftopoulou S. Kienzl M. Santiso A. (2021). Identification of novel low-density neutrophil markers through unbiased high-dimensional flow cytometry screening in non-small cell lung cancer patients. Front. Immunol. 12, 703846. 10.3389/fimmu.2021.703846 34484199 PMC8414579

[B99] Veglia F. Tyurin V. A. Blasi M. De Leo A. Kossenkov A. V. Donthireddy L. (2019). Fatty acid transport protein 2 reprograms neutrophils in cancer. Nature 569, 73–78. 10.1038/s41586-019-1118-2 30996346 PMC6557120

[B100] Wang C. Zheng X. Zhang J. Jiang X. Wang J. Li Y. (2023a). CD300ld on neutrophils is required for tumour-driven immune suppression. Nature 621, 830–839. 10.1038/s41586-023-06511-9 37674079

[B101] Wang J. Wang X. Guo Y. Ye L. Li D. Hu A. (2021). Therapeutic targeting of SPIB/SPI1‐facilitated interplay of cancer cells and neutrophils inhibits aerobic glycolysis and cancer progression. Clin. Transl. Med. 11, e588. 10.1002/ctm2.588 34841706 PMC8567044

[B102] Wang L. Liu Y. Dai Y. Tang X. Yin T. Wang C. (2023b). Single-cell RNA-seq analysis reveals BHLHE40-driven pro-tumour neutrophils with hyperactivated glycolysis in pancreatic tumour microenvironment. Gut 72, 958–971. 10.1136/gutjnl-2021-326070 35688610 PMC10086491

[B103] Wang P. Yang G. L. He Y. F. Shen Y. H. Hao X. H. Liu H. P. (2024). Single-cell transcriptomics of blood identified IFIT1(+) neutrophil subcluster expansion in NTM-PD patients. Int. Immunopharmacol. 137, 112412. 10.1016/j.intimp.2024.112412 38901242

[B104] Wang Y. Xu M. Sun J. Li X. Shi H. Wang X. (2023c). Glycolytic neutrophils accrued in the spleen compromise anti-tumour T cell immunity in breast cancer. Nat. Metab. 5, 1408–1422. 10.1038/s42255-023-00853-4 37563468

[B105] Watts E. R. Howden A. J. Morrison T. Sadiku P. Hukelmann J. Von Kriegsheim A. (2021). Hypoxia drives murine neutrophil protein scavenging to maintain central carbon metabolism. J. Clin. Invest 131, e134073. 10.1172/JCI134073 33822765 PMC8121528

[B106] Wculek S. K. Malanchi I. (2015). Neutrophils support lung colonization of metastasis-initiating breast cancer cells. Nature 528, 413–417. 10.1038/nature16140 26649828 PMC4700594

[B107] Wellenstein M. D. De Visser K. E. (2019). Fatty acids corrupt neutrophils in cancer. Cancer Cell 35, 827–829. 10.1016/j.ccell.2019.05.007 31185209

[B108] Wu Y. Ma J. Yang X. Nan F. Zhang T. Ji S. (2024). Neutrophil profiling illuminates anti-tumor antigen-presenting potency. Cell 187, 1422–1439.e24. 10.1016/j.cell.2024.02.005 38447573

[B109] Xiong T. He P. Zhou M. Zhong D. Yang T. He W. (2022a). Glutamate blunts cell-killing effects of neutrophils in tumor microenvironment. Cancer Sci. 113, 1955–1967. 10.1111/cas.15355 35363928 PMC9207372

[B110] Xiong T. He P. Zhou M. Zhong D. Yang T. He W. (2022b). Glutamate blunts cell‐killing effects of neutrophils in tumor microenvironment. Cancer Sci. 113, 1955–1967. 10.1111/cas.15355 35363928 PMC9207372

[B111] Yang S. Chen J. Xie K. Liu F. (2024). NPC1 promotes the progression of hepatocellular carcinoma by mediating the accumulation of neutrophils into the tumor microenvironment. FEBS Open Bio 15, 661–673. 10.1002/2211-5463.13951 PMC11961396 39707615

[B112] Yang X. Lu Y. Hang J. Zhang J. Zhang T. Huo Y. (2020). Lactate-modulated immunosuppression of myeloid-derived suppressor cells contributes to the radioresistance of pancreatic cancer. Cancer Immunol. Res. 8, 1440–1451. 10.1158/2326-6066.CIR-20-0111 32917658

[B113] Yan L. Wu M. Wang T. Yuan H. Zhang X. Zhang H. (2024). Breast cancer stem cells secrete MIF to mediate tumor metabolic reprogramming that drives immune evasion. Cancer Res. 84, 1270–1285. 10.1158/0008-5472.CAN-23-2390 38335272

[B114] Zeng Z. Xu S. Wang F. Peng X. Zhang W. Zhan Y. (2022). HAO1-mediated oxalate metabolism promotes lung pre-metastatic niche formation by inducing neutrophil extracellular traps. Oncogene 41, 3719–3731. 10.1038/s41388-022-02248-3 35739335 PMC9287177

[B115] Zhang H. Zhu X. Friesen T. J. Kwak J. W. Pisarenko T. Mekvanich S. (2022a). Annexin A2/TLR2/MYD88 pathway induces arginase 1 expression in tumor-associated neutrophils. J. Clin. Invest 132, e153643. 10.1172/JCI153643 36377658 PMC9663166

[B116] Zhang H. Zhu X. Friesen T. J. Kwak J. W. Pisarenko T. Mekvanich S. (2022b). Annexin A2/TLR2/MYD88 pathway induces arginase 1 expression in tumor-associated neutrophils. J. Clin. Investigation 132, e153643. 10.1172/JCI153643 PMC9663166 36377658

[B117] Zhang J. Xu X. Shi M. Chen Y. Yu D. Zhao C. (2017). CD13(hi) Neutrophil-like myeloid-derived suppressor cells exert immune suppression through Arginase 1 expression in pancreatic ductal adenocarcinoma. Oncoimmunology 6, e1258504. 10.1080/2162402X.2016.1258504 28344866 PMC5353902

[B118] Zhang J. Yu D. Ji C. Wang M. Fu M. Qian Y. (2024). Exosomal miR-4745-5p/3911 from N2-polarized tumor-associated neutrophils promotes gastric cancer metastasis by regulating SLIT2. Mol. Cancer 23, 198. 10.1186/s12943-024-02116-6 39272149 PMC11396805

[B119] Zhang Z. Zheng Y. Chen Y. Yin Y. Chen Y. Chen Q. (2022c). Gut fungi enhances immunosuppressive function of myeloid-derived suppressor cells by activating PKM2-dependent glycolysis to promote colorectal tumorigenesis. Exp. Hematol. Oncol. 11, 88. 10.1186/s40164-022-00334-6 36348389 PMC9644472

[B120] Zhao Y. Liu Z. Liu G. Zhang Y. Liu S. Gan D. (2023). Neutrophils resist ferroptosis and promote breast cancer metastasis through aconitate decarboxylase 1. Cell Metab. 35, 1688–1703.e10. 10.1016/j.cmet.2023.09.004 37793345 PMC10558089

[B121] Zhou J. Nefedova Y. Lei A. Gabrilovich D. (2018). Neutrophils and PMN-MDSC: their biological role and interaction with stromal cells. Semin. Immunol. 35, 19–28. 10.1016/j.smim.2017.12.004 29254756 PMC5866202

[B122] Zhou J. Xu W. Wu Y. Wang M. Zhang N. Wang L. (2023). GPR37 promotes colorectal cancer liver metastases by enhancing the glycolysis and histone lactylation via Hippo pathway. Oncogene 42, 3319–3330. 10.1038/s41388-023-02841-0 37749229

[B123] Zhu D. Lu Y. Yan Z. Deng Q. Hu B. Wang Y. (2024). A β-carboline derivate PAD4 inhibitor reshapes neutrophil phenotype and improves the tumor immune microenvironment against triple-negative breast cancer. J. Med. Chem. 67, 7973–7994. 10.1021/acs.jmedchem.4c00030 38728549

[B124] Zhu H. Yu H. Zhou H. Zhu W. Wang X. (2023). Elevated nuclear PHGDH synergistically functions with cMyc to reshape the immune microenvironment of liver cancer. Adv. Sci. 10, e2205818. 10.1002/advs.202205818 PMC10265107 37078828

